# FUS pathology in ALS is linked to alterations in multiple ALS-associated proteins and rescued by drugs stimulating autophagy

**DOI:** 10.1007/s00401-019-01998-x

**Published:** 2019-04-01

**Authors:** Lara Marrone, Hannes C. A. Drexler, Jie Wang, Priyanka Tripathi, Tania Distler, Patrick Heisterkamp, Eric Nathaniel Anderson, Sukhleen Kour, Anastasia Moraiti, Shovamayee Maharana, Rajat Bhatnagar, T. Grant Belgard, Vadreenath Tripathy, Norman Kalmbach, Zohreh Hosseinzadeh, Valeria Crippa, Masin Abo-Rady, Florian Wegner, Angelo Poletti, Dirk Troost, Eleonora Aronica, Volker Busskamp, Joachim Weis, Udai Bhan Pandey, Anthony A. Hyman, Simon Alberti, Anand Goswami, Jared Sterneckert

**Affiliations:** 1 Technische Universität Dresden, Center for Regenerative Therapies Dresden, Fetscherstr. 105, 01307 Dresden, Germany; 2 Max Planck Institute for Molecular Biomedicine, Bioanalytical Mass Spectrometry, Röntgenstr. 20, 48149 Münster, Germany; 3 Max Planck Institute of Molecular Cell Biology and Genetics, Pfotenhauerstr. 108, 01307 Dresden, Germany; 4 Institute of Neuropathology, RWTH Aachen University Hospital, Pauwelsstr. 30, 52074 Aachen, Germany; 5 Division of Child Neurology, Department of Pediatrics, Children’s Hospital of Pittsburgh, University of Pittsburgh School of Medicine, Pittsburgh, PA USA; 6 Department of Human Genetics, University of Pittsburgh Graduate School of Public Health, Pittsburgh, PA USA; 7 Verge Genomics, San Francisco, CA USA; 8 Department of Neurology, Hannover Medical School, Carl-Neuberg-Str. 1, 30625 Hannover, Germany; 9 Dipartimento di Scienze Farmacologiche e Biomolecolari, Centre of Excellence on Neurodegenerative Diseases Università degli studi di Milano, Milan, 20133 Italy; 10 Department of (Neuro)Pathology, Amsterdam Neuroscience, Amsterdam UMC, University of Amsterdam, Amsterdam, The Netherlands; 11 Department of Neurology, University of Pittsburgh School of Medicine, Pittsburgh, PA USA; 12 Present Address: The Bioinformatics CRO, Niceville, FL USA

**Keywords:** Amyotrophic lateral sclerosis, FUS, Induced pluripotent stem cells, RNA-binding proteins, Phase transition, Protein homeostasis

## Abstract

Amyotrophic lateral sclerosis (ALS) is a lethal disease characterized by motor neuron degeneration and associated with aggregation of nuclear RNA-binding proteins (RBPs), including FUS. How FUS aggregation and neurodegeneration are prevented in healthy motor neurons remain critically unanswered questions. Here, we use a combination of ALS patient autopsy tissue and induced pluripotent stem cell-derived neurons to study the effects of FUS mutations on RBP homeostasis. We show that FUS’ tendency to aggregate is normally buffered by interacting RBPs, but this buffering is lost when FUS mislocalizes to the cytoplasm due to ALS mutations. The presence of aggregation-prone FUS in the cytoplasm causes imbalances in RBP homeostasis that exacerbate neurodegeneration. However, enhancing autophagy using small molecules reduces cytoplasmic FUS, restores RBP homeostasis and rescues motor function in vivo. We conclude that disruption of RBP homeostasis plays a critical role in FUS-ALS and can be treated by stimulating autophagy.

**Electronic supplementary material:**

The online version of this article (10.1007/s00401-019-01998-x) contains supplementary material, which is available to authorized users.

## Introduction

Amyotrophic lateral sclerosis (ALS) is the most common motor neuron (MN) disease [28]. ALS pathology is characterized by preferential degeneration of upper and lower motor neurons, which leads to progressive paralysis and eventually death. Since available treatments do not effectively prevent or slow disease progression, novel therapeutics are urgently needed.

There is increasing evidence that defects in the homeostasis of RBPs, i.e. changes in their expression levels or subcellular localization, are critically involved in driving the onset of ALS. Approximately 10% of all ALS cases are familial, many of which have been linked to genetic mutations in RNA-binding proteins (RBPs), including Fused in sarcoma (FUS), TAR DNA-binding protein 43 (TDP43), Matrin3, Ewing sarcoma breakpoint region 1 (EWSR1), TATA-Box Binding Protein Associated Factor 15 (TAF15), heterogeneous nuclear ribonucleoprotein A1 (hnRNPA1), hnRNPA2B1, and TIA1 [15]. These RBPs are structurally related, containing at least one RNA-recognition motif (RRM), a nuclear localization signal (NLS), and a low complexity domain, which is required for phase separation. ALS-associated mutations accelerate an aberrant liquid-to-solid phase transition of these proteins [21], and aggregates containing RBPs have been detected in ALS patient MNs.

ALS patients with mutant *FUS* exhibit a particularly severe progression, with 60% of cases developing pathology before 40 years of age. *FUS* mutations primarily occur in the NLS domain, such as P525L and R521C, inducing FUS cytoplasmic mislocalization due to reduced interactions with the nuclear import receptor Transportin-1 [6]. However, many questions remain: How do healthy motor neurons maintain FUS homeostasis? How is FUS homeostasis disrupted by ALS-causing FUS mutations in the NLS? Do FUS mutations affect other ALS-associated RBPs? How can the function of RBPs such as FUS be restored? Do the various ALS-associated RBP mutations each require a specific treatment? Answering these questions should contribute to the development of effective therapeutics for ALS patients.

In this manuscript, we use a combination of neuropathology and induced pluripotent stem cell (iPSC)-derived neurons to study the effects of FUS mutations on protein homeostasis. We show that MNs in spinal cord tissue from ALS patients with mutant *FUS* express heterogeneous levels of cytoplasmic FUS protein, and we use gene-edited WT and P525L FUS-eGFP iPSCs to model this heterogeneity. We demonstrate that the cytoplasmic mislocalization caused by mutations in the FUS NLS impairs the interaction of FUS with other ALS-associated RBPs. Purified hnRNPA1, EWSR1, and TAF15 prevent FUS from undergoing an aberrant liquid-to-solid phase transition, indicating that FUS mislocalization disrupts FUS interactions with these RBPs, facilitating the nucleation of toxic cytoplasmic FUS aggregates. We additionally show that iPSC-derived neurons with high cytoplasmic FUS levels exhibit defects in protein degradation, which is marked by increased p62, as well as reduced protein levels of hnRNPA1, hnRNPA2B1, EWSR1, and TAF15, which we confirm using human autopsy tissue. Knocking down hnRNPA1, hnRNPA2B1, EWSR1, and TAF15 induces neurodegeneration, thus highlighting their importance for neuronal viability. Finally, we show that small molecules inducing autophagy restore homeostasis of all misregulated proteins and ameliorate motor function in vivo. Taken together, our results establish that FUS-ALS pathology is mechanistically linked to the homeostasis of multiple ALS-associated RBPs, which can be ameliorated by drugs inducing autophagy. Since impaired homeostasis is a hallmark of multiple ALS subtypes, drugs inducing autophagy could be effective therapeutics for many ALS patients.

## Materials and methods

See Online Resource 1 for a complete description of all materials and methods.

### Ethical approval

All procedures involving human participants were performed in accordance with the ethical standards of the institutional and/or national research committee as well as with the 1964 Helsinki declaration and its later amendments.

### Cell culture and treatments

iPSC-derived cell lines used in this study were previously characterized and cultured as described [18]. Sodium arsenite (0.5 mM, Fluka) and cycloheximide (100 µg/ml, Sigma-Aldrich) were added for 1 h. All the other treatments, including torkinib (10 µM, Selleckchem), PQR309 (10 µM, Medchem), Adox (100 µM, Santa Cruz), MG132 (5 µM, Selleckchem), 3-MA (2.5 mM, Selleckchem) were performed over 24 h or 48 h. Lentivectors for shRNA-mediated knockdown were generated in house as described in the Supplements. 90 k neurons were infected at day 10 of maturation with lentiviral particles in the presence of 10 µg/ml protamine sulfate (Sigma-Aldrich). Cells were analyzed after another 9 days from transduction.

### Protein analysis

Cells were lysed in RIPA buffer (Santa Cruz Biotechnology). Lysates were analyzed by capillary electrophoresis using the Protein Simple WES™ 12-230 Separation Module. FUS-eGFP was immunoprecipitated using a GFP-Trap^®^ Kit (Chromotek). Label-free mass spectrometric analysis of immunoprecipitated proteins was performed as described in the extended methods section attached as Supplementary information.

### Drosophila experiments

All *Drosophila* stocks were maintained on standard cornmeal at 25 °C in light/dark-controlled incubators. The w1118, UAS-eGFP, and D42-GAL4 were obtained from the Bloomington stock center. The UAS-FUS WT, UAS-FUS P525L, and UAS-FUS R521C flies as well as the experimental conditions for climbing index assessment were previously described [1].

### Patient samples and staining

Human post-mortem brain and spinal cord samples (*n* = 6 age-matched controls, *n* = 6 R521C FUS mutation) were obtained from the Department of (Neuro-)Pathology, Academic Medical Center (AMC), University of Amsterdam. All tissues were fixed in buffered formalin within 6–24 h from death. 3–4 µm paraffin sections were processed for either immunohistochemistry (DAB and haematoxylin) or immunofluorescent staining as previously described [7, 11]. Primary antibodies included: rabbit anti-FUS (AMAB90549, Sigma) 1:150, mouse anti-hnRNPA1 (NB100-672, Novusbio) 1:200, mouse anti-hnRNPA2B1 (sc-32316, Santa Cruz) 1:200. Images were acquired with either a Zeiss Axioplan microscope for immunohistochemistry, or a Zeiss LSM 700 laser scanning confocal microscope for immunofluorescence.

## Results

### Heterogeneity in MN populations in ALS-patient spinal cord is recapitulated by FUS-eGFP iPSC-derived neurons with different linker lengths

**Fig. 1:**
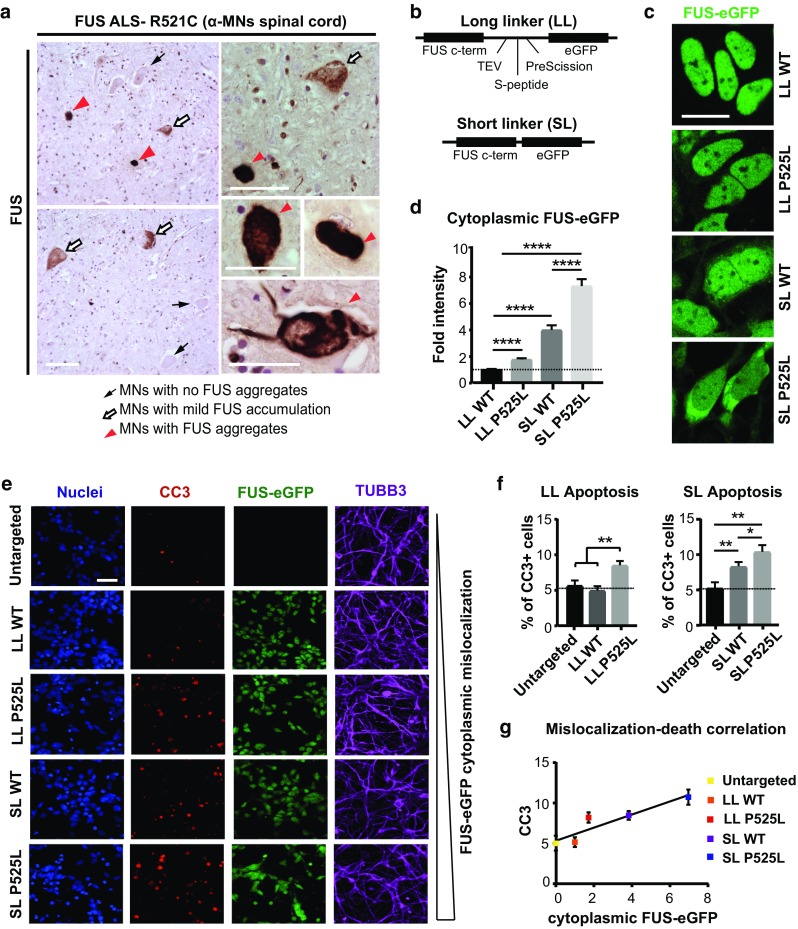
FUS-eGFP lines used in this study recapitulate ALS pathology observed in patients. **a** FUS immunoreactivity of human lumbar spinal cord α-MNs of FUS patients (*n* = 6). Intensely labelled globular inclusions (red arrowheads), diffuse cytoplasmic accumulations (white arrows), normal nuclear FUS immunolabelling (black arrows). Scale bars = 60 μM. **b** Schematic representation of the linkers used to tag endogenous *FUS* with eGFP. **c** Confocal micrographs showing FUS-eGFP subcellular localization in the iPSC-derived neurons used in this study. Scale bar = 10 µm. **d** Quantification of cytoplasmic FUS-eGFP fluorescence intensity in the iPSC-derived neurons shown in **c**. **e** Immunofluorescence staining for the indicated markers. **f** Apoptosis in SL and LL FUS-eGFP neurons is quantified as percentage of cleaved Caspase 3 (CC3)-positive cells. *n* = 3, and for each independent experiment 3 confocal images were analyzed. Scale bar 50 µm. **g** Apoptosis in iPSC-derived neurons correlates with the amount of cytoplasmic FUS-eGFP. Linear correlation was calculated using Pearson’s correlation. *R*^2^ = 0.859 Error bars represent standard error of the mean (SEM). * and ** Correspond to *p* < 0.05 and *p* < 0.01, respectively

A defining trait of ALS is the progression of motor dysfunction. Although disease onset is typically focal, symptoms rapidly spread in a manner that reflects the organization of the underlying neuronal circuitry, usually in a corticofugal fashion [3, 4]. Although ALS-causing mutations affect genes, such as FUS, that are ubiquitously and constitutively expressed, MNs appear to be particularly vulnerable. Fast-twitch fatigable α-MNs are among the first to degenerate, whereas other MN subtypes degenerate in later stages of the disease [22]. Thus, MNs do not degenerate synchronously, and understanding the properties that confer vulnerability to some neurons and resistance to others may have profound therapeutic value. When we immunostained human spinal cord tissue from ALS patients carrying the FUS-NLS mutation R521C, we found that the lumbar spinal cord of FUS-ALS cases showed severe loss of α-MNs [suppl. Figure 1 (Online Resource 2)], and the surviving α-MNs displayed varying degrees of FUS protein mislocalization and cytoplasmic accumulation [Fig. 1a; suppl. Figure 1b (Online Resource 2)]. Thus, MNs of ALS patients show heterogeneous levels of FUS neuropathology with some MNs showing higher levels of FUS accumulation than others.

To better understand the molecular pathomechanisms driving FUS-ALS, we previously generated isogenic iPSC reporter lines with WT and P525L FUS-eGFP capable of differentiating into electrically active neurons [suppl. Figure 2 (Online Resource 2)], which consisted of a mixed population of spinal neurons containing 15% of *bona fide* MNs [suppl. Figure 3 (Online Resource 2)] [18]. To tag the *FUS* c-terminus of only one allele with eGFP (Fig. 1b), we used two different linkers, which produced distinct effects on the subcellular localization of the FUS protein. Using a long linker (LL), which was previously characterized, we found that WT FUS-eGFP was exclusively localized in the nucleus (Fig. 1c, d). In contrast, isogenic iPSC-derived neurons with P525L FUS-eGFP showed a two-fold increase in cytoplasmic FUS-eGFP fluorescent intensity (Fig. 1c, d), demonstrating that P525L induces FUS mislocalization. However, FUS-eGFP mislocalization in neurons carrying the P525L mutation in the presence of the LL was only minor. Since patient MNs are heterogeneous with some showing prominent FUS pathology, we also used a shorter linker stretch (SL), which partially interfered with the function of the adjacent NLS domain (Fig. 1b). For this reason, the SL caused a basal level of FUS-eGFP mislocalization, which was 4 times higher than in LL lines (Fig. 1c, d). In particular, SL P525L iPSC-derived neurons showed the highest amount of cytoplasmic FUS protein, manifesting an 8-fold increase compared to LL WT neurons (Fig. 1c, d). Thus, we used these lines to recapitulate the heterogeneity found in patients and investigate the impact of differential FUS mislocalization on the progression of the degenerative process.

As ALS is characterized by neurodegeneration, we immunostained differentiated neurons for cleaved-Caspase 3 (CC3), which marks apoptosis (Fig. 1e). We observed that P525L significantly increased apoptosis in neurons with LL as well as SL (Fig. 1f). In addition, we observed that CC3 levels correlated well with the amount of cytoplasmic FUS-eGFP (Fig. 1g). Indeed, neurons with SL P525L FUS-eGFP showed the highest levels of cell death (Fig. 1f, g). These data supported a link between cytoplasmic FUS-eGFP levels and neurodegeneration, confirming that our iPSC-derived spinal neurons represent a suitable model to recapitulate the heterogeneity observed in FUS-ALS patients.

### FUS-ALS is linked to defects in protein quality control

**Fig. 2:**
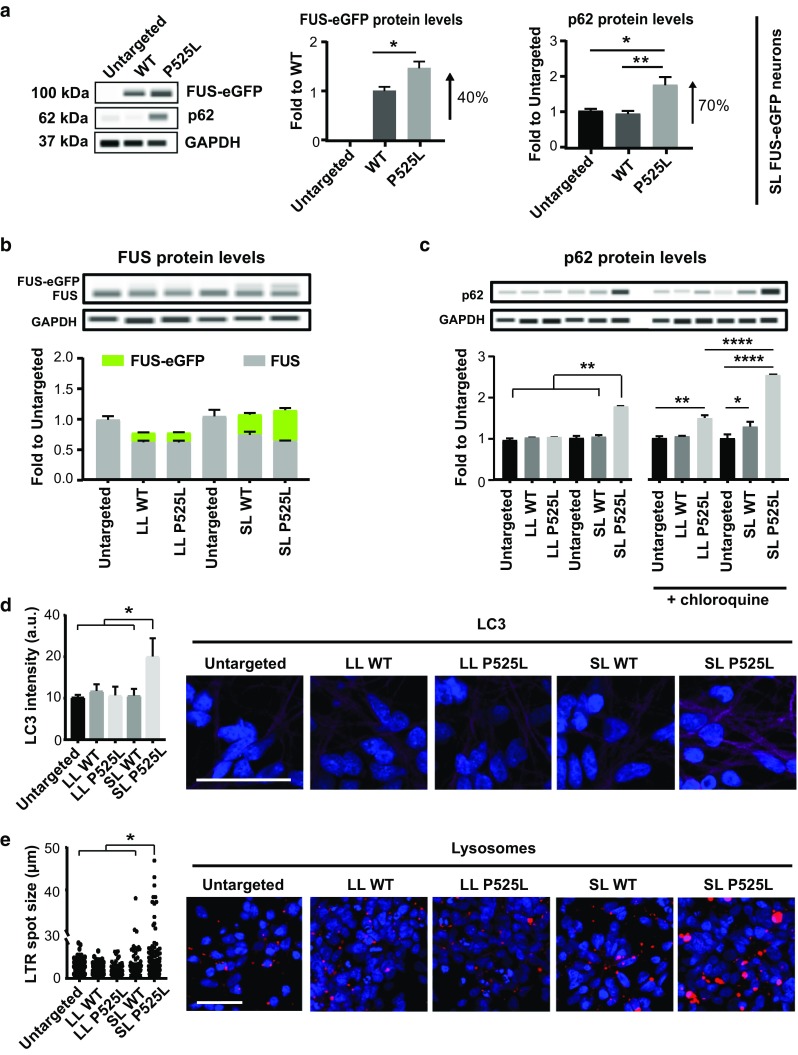
Protein clearance in FUS iPSC-derived neurons. **a** WES capillary electrophoresis and respective quantification of FUS-eGFP and p62 proteins in SL iPSC-derived neurons. **b** Overview of FUS and FUS-eGFP protein levels using WES capillary electrophoresis. **c** Quantification of p62 protein levels in basal conditions and following 50 µm chloroquine treatment for 24 h in the differentiated neurons with the indicated genotype. **d** Confocal immunofluorescent images and quantification of LC3 using neurons with the indicated genotype. **e** Confocal fluorescent images and quantification of lysosomes using neurons with the indicated genotype. Scale bar 50 µm. *n* = 3. Error bars indicate standard deviation. *, **, and **** Correspond to *p* < 0.05, *p* < 0.01 and *p* < 0.0001, respectively

Because impaired FUS homeostasis has been linked to ALS pathology, we used capillary electrophoresis to quantify total FUS protein levels (Fig. 2a, b). The results showed a significant accumulation of FUS-eGFP protein specifically in iPSC-derived neurons with SL P525L (Fig. 2a). This observation led us to speculate that heterogeneity in FUS pathology could be linked to differences in protein degradation. To test this hypothesis, we evaluated p62 protein levels, which typically increase the following defects in proteasome activity and autophagy. We found that LL WT-eGFP, LL P525L FUS-eGFP and SL WT FUS-eGFP neurons had p62 levels comparable to neurons from non-transgenic parental iPSCs [Fig. 2a; suppl. Figure 4a (Online Resource 2)]. In contrast, SL P525L FUS-eGFP neurons exhibited almost two-fold more p62 than WT (Fig. 2a). Thus, neurons with SL P525L FUS-eGFP not only presented the highest levels of cytoplasmic FUS, but they also displayed increased FUS-eGFP as well as p62 levels, suggesting defects in protein degradation. Using immunofluorescence, we observed that p62 was diffused throughout the soma of SL P525L neurons with its intensity occasionally increasing in subcellular localizations with FUS accumulation [suppl. Figure 4b, c (Online Resource 2)]. The correlation of p62 and FUS became more evident in arsenite-stressed cells, in which a subset of FUS-eGFP stress granules (SGs) clearly co-localized with p62 assemblies, suggesting that p62 interacts with FUS, and its accumulation is linked to the increment in FUS-eGFP levels [suppl. Figure 4d (Online Resource 2)].

Next, we looked at autophagy in our cultures. Consistent with our earlier observations, combined inhibition of autophagy and proteasome activity in LL P525L neurons using 3-MA + MG132 treatment resulted in FUS-eGFP being strongly mislocalized and reaching cytoplasmic levels similar to those of untreated SL P525L neurons [suppl. Figure 4a, c (Online Resource 2)]. This was accompanied by a significant increase in the overall levels of both FUS-eGFP and p62 [suppl. Figure 4a (Online Resource 2)], confirming a critical connection between cytoplasmic FUS levels and protein homeostasis. Importantly, when we blocked the autophagic flux using chloroquine, we also observed an increase of p62 (Fig. 2c) and LC3-II [suppl. Figure 4e (Online Resource 2)] in all neuronal cultures with mislocalized FUS-eGFP. This suggested that an impairment in autophagy alone without inhibiting proteasome activity is sufficient to induce the pathological increase in p62 levels. Investigating basal autophagy in our cultures, we found that, in addition to showing increased p62, SL P525L neurons had significantly increased steady-state levels of LC3 (Fig. 2d) as well as strongly enlarged lysosomes (Fig. 2e). Such morphology was previously associated with aberrant lysosomal dynamics [8], suggesting that ALS neurons with high cytoplasmic FUS levels exhibit impaired autophagy. In conclusion, we demonstrated that FUS ALS is characterized by a tight interplay between autophagic clearance, p62, and cytoplasmic FUS protein levels.

### P525L disrupts FUS interactions with other ALS-associated RBPs

**Fig. 3:**
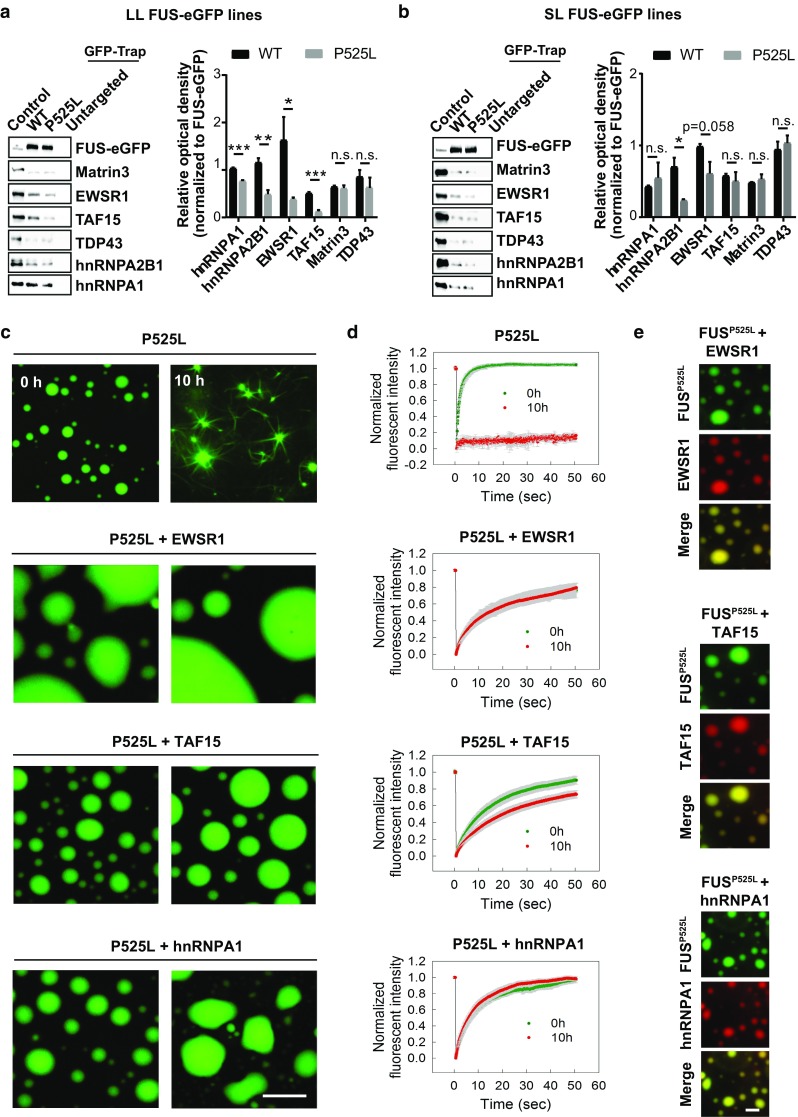
The cytoplasmic mislocalization induced by P525L causes reduced FUS binding to several ALS-associated RBPs, promoting aggregation. **a**, **b** Western blot analysis of FUS protein interactors in **a** LL and **b** SL neurons after FUS-eGFP immunoprecipitation reveals differential interactions with several ALS-associated partners. *n* = 4. Error bars indicate SEM. *, **, and *** Correspond to *p* < 0.05, 0.01, and 0.001, respectively. **c** In vitro phase separation assay showing fibrillization of purified P525L LL FUS-eGFP protein in the presence or absence of distinct RBPs. Investigated RBPs effectively prevent FUS fibril formation. **d** Fluorescence recovery after photobleaching (FRAP) was used to assess the dynamics of P525L LL FUS at the tested conditions for the indicated time points. RBPs promote the maintenance of a liquid-like behavior. **e** Co-localization of P525L LL FUS with the reported RBPs. Scale bar 5 µm

We hypothesized that the mislocalization of FUS to the cytoplasm would alter FUS protein–protein interactions and that some of the affected interactions would be integral to the induction of ALS. To test this, we exploited the eGFP reporter sequence as an affinity tag for selective immunoprecipitation of FUS-eGFP, followed by analysis of the FUS interactome by liquid chromatography coupled to tandem mass spectrometry (LC–MS/MS). To dissect the impact of the FUS cytoplasmic shift, we focused on LL lines and compared results from LL WT FUS-eGFP neurons, which exhibit exclusive nuclear localization, with isogenic LL P525L FUS-eGFP cultures. Neurons from the non-transgenic parental line were also included to filter out non-specific interactors. In this experiment, we identified approximately 200 proteins as potential FUS interactors [suppl. Table 1 (Online Resource 3)], and gene ontology analysis showed enrichment for RNA, ribosomal and spliceosomal complexes [suppl. Table 2 (Online Resource 4)], which is consistent with the known functions of FUS. When we specifically focused on ALS-associated proteins, we found that a number of FUS interaction partners encompassed RBPs previously described to cause familial forms of ALS, including hnRNPA1, hnRNPA2B1, EWSR1 and TAF15, were reduced in pulldown samples with P525L FUS compared to WT. Immunoblot confirmed the LC–MS/MS results and showed significantly decreased interactions of P525L FUS with hnRNPA1, hnRNPA2B1, EWSR1 and TAF15, while binding to other RBPs, such as TDP43 and MATR3, remained unchanged (Fig. 3a). We then extended our quantification to SL neurons, where hnRNPA2B1 again showed significantly reduced levels in P525L pulldowns compared to WT (Fig. 3b). Although this did not reach statistical significance, hnRNPA1, TAF15 and EWSR1 exhibited a trend towards reduced interactions with P525L FUS (Fig. 3b). It is likely that differences in FUS interactions with RBPs were difficult to detect in SL neurons because SL WT and P525L FUS-eGFP are both mislocalized, thus negatively impacting on RBP enrichment in both cases. Taken together, these results demonstrate that P525L reduces the interaction between FUS and certain ALS-associated RBPs.

### ALS-associated RBPs inhibit liquid-to-solid phase transition of FUS protein

Protein solubility plays a critical role in FUS function and ALS pathogenesis [21]. Phase separation of FUS into membraneless compartments is required for DNA damage repair, mRNA transport, as well as SG formation. In addition, phase separation could seed the formation of aggregates in ALS by promoting an aberrant liquid-to-solid phase transition. It was previously shown that both Transportin-1 and nuclear RNAs can modulate FUS phase separation behavior [9, 17]. Therefore, we sought to determine whether the interaction of FUS with other RBPs may similarly impact on the dynamics of FUS liquid-to-solid phase transition. To test this, we performed an in vitro phase separation assay where we aged LL P525L FUS for 10 h in the presence or absence of RBPs (Fig. 3c). Fluorescence recovery after photobleaching (FRAP) showed that P525L FUS droplets analyzed at 0 h of age recovered rapidly, consistent with them being liquid (Fig. 3d). However, after 10 h of aging, P525L FUS had acquired a fibril-like morphology and was unable to recover, which suggested the formation of solid aggregates (Fig. 3c, d). Importantly, when P525L FUS droplets were aged in the presence of EWSR1, TAF15 and hnRNPA1, fiber formation was suppressed (Fig. 3c, d). P525L FUS interaction with the investigated RBPs was confirmed by assessing droplet co-localization (Fig. 3e). Taken together, these data demonstrated that the investigated ALS-associated RBPs can modulate the phase behavior of FUS and inhibit aggregation. We conclude that the cytoplasmic shift induced by FUS NLS mutations reduces the interaction of FUS with nuclear RBPs, facilitating FUS aggregation.

### ALS-associated RBPs are post-transcriptionally reduced in neurons with SL P525L FUS-eGFP

**Fig. 4:**
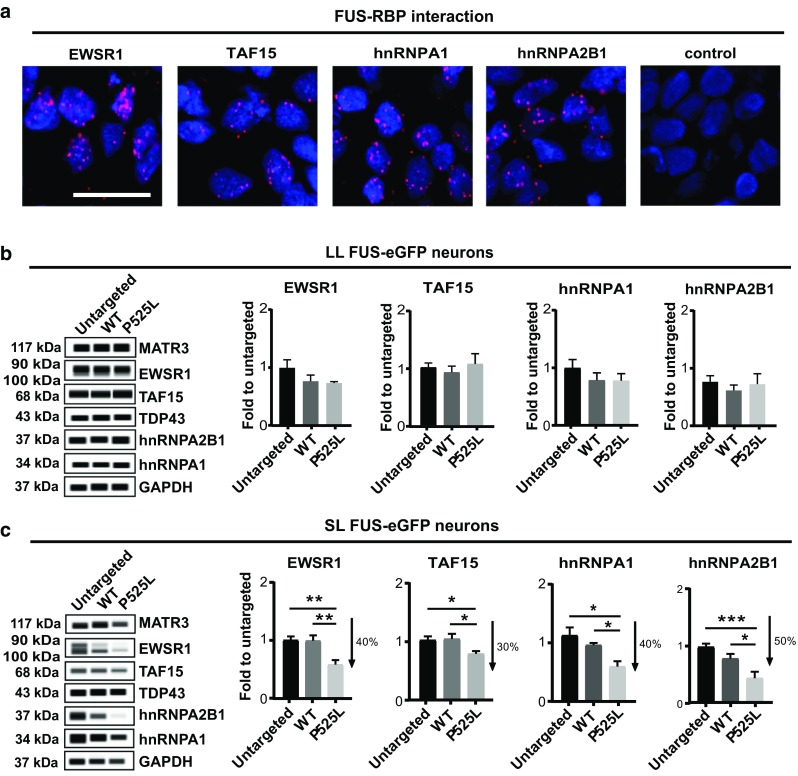
Relationship between FUS and the identified ALS-associated RBPs. **a** Proximity ligation assay confirms FUS interaction with EWSR1, TAF15, hnRNPA1 and hnRNPA2B1 in iPSC-derived neurons. Protein–protein interactions appear as distinct spots. Scale bar = 25 µm. **b, c** WES capillary electrophoresis for quantification of the overall cellular levels of the RBPs found to interact with FUS in LL **b**, **c** SL neurons. Analysis reveals a significant decrease in EWSR1, TAF15, hnRNPA1 and hnRNPA2B1 levels, which is specific to P525L FUS SL neurons. *n* = 5. Error bars indicate SEM. *, **, and *** Correspond to *p* < 0.05, 0.01, and 0.001, respectively

Since FUS interacts with a number of ALS-associated RBPs (Fig. 4a) and functions as a protein complex, we speculated that high levels of cytoplasmic FUS might affect the protein levels of other ALS-associated RBPs. To test this, we used capillary electrophoresis to quantify their amounts in whole neuronal lysates. The investigated RBPs did not display any significant change between WT and P525L genotypes in LL neuronal cultures (Fig. 4b), confirming that the differences in FUS association were due to FUS mislocalization. Notably, when we analyzed SL cultures, we observed a significant reduction of EWSR1, TAF15, hnRNPA1 and hnRNPA2B1 selectively in SL P525L neurons compared to WT (Fig. 4c). This demonstrates that high levels of cytoplasmic FUS in neurons lead to a reduction in the levels of certain ALS-associated RBPs.

ALS is a neurodegenerative disease, which implies that, although mutant FUS is ubiquitously expressed, ALS pathology has a certain degree of cell-type specificity. For this reason, we tested the specificity of reduced levels of EWSR1, TAF15, hnRNPA1 and hnRNPA2B1. To do this, we quantified their levels by performing immunoblotting using undifferentiated iPSCs as well as neural progenitors (NPCs). In contrast to differentiated neurons, iPSCs and iPSC-derived NPCs showed no differences in the levels of EWSR1, TAF15, hnRNPA1 and hnRNPA2B1 [suppl. Figure 5a, c (Online Resource 2)]. Consistent with this observation, p62 levels were also unchanged in iPSCs and iPSC-derived NPCs [(suppl. Figure 5b (Online Resource 2)], highlighting the significance of studying ALS in disease-relevant cell types, such as human spinal neurons.

Because FUS levels have been suggested to modulate the mRNA expression of EWSR1, TAF15 and hnRNPA1 [12], we asked whether observed changes in protein levels would depend on transcriptional regulation. To test this, we performed RNA profiling on SL WT and P525L neurons, including the untargeted parental line as a reference. Interestingly, we observed no significant differences in the expression levels of EWSR1, TAF15, hnRNPA1, and hnRNPA2B1 across genotypes [suppl. Table 3 (Online Resource 5)]. We concluded that the reduction in the levels of the investigated ALS-associated RBPs in P525L SL FUS-eGFP neurons occurs at a post-transcriptional level.

### Levels of ALS-associated RBPs inversely correlate with FUS aggregation in ALS patient MNs

**Fig. 5:**
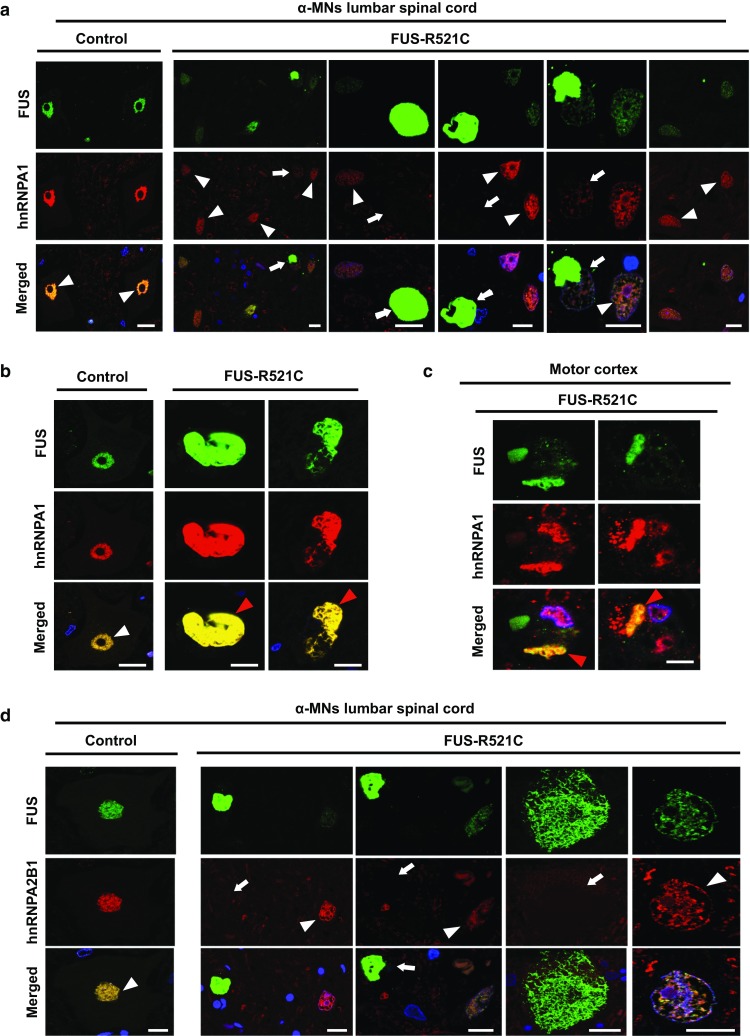
Immunohistochemistry of human tissue from FUS-ALS patients. **a**–**d** Double immunolabelling using FUS antibody together with **a**–**c** hnRNPA1 and **d** hnRNPA2B1 in human FUS-ALS lumbar spinal cord compared to normal age-matched controls. *n* = 6. Nuclear hnRNPA1and FUS immunoreactivity in normal control α-MNs (white arrow head, left panel). Surviving α-MNs in FUS-R521C cases containing FUS aggregates show low hnRNPA1 levels (white arrows), while MNs without FUS aggregates display normal levels (white arrowheads). Normal nuclear co-localization of hnRNPA1 with FUS (white arrowhead) in a control case; rare co-localization of cytoplasmic hnRNPA1 accumulations with FUS aggregates (red arrowhead) in **b** α-MNs and **c** motor cortex. Scale bars 15 µm

Next, we sought to relate our findings to the neuropathological alterations in FUS-ALS patients [suppl. Table 4 (Online Resource 6)]. In iPSC-derived spinal neurons, as described above, we observed that FUS NLS mutations, such as P525L, result in FUS protein accumulation, decreased interaction with ALS-associated RBPs, and reduced levels of these RBPs. Since the investigated RBPs protected purified FUS from fibrillization, we hypothesized that their levels would inversely correlate with the presence of FUS aggregates in ALS patient MNs. To test this, we used autopsy tissue (lumbar spinal cord and/or motor cortex) from FUS-ALS patients and performed double immunolabelling using antibodies against FUS and our target RBPs. Surviving α-MNs harboring FUS inclusions showed reduced levels of hnRNPA1 and hnRNPA2B1 compared to α-MNs lacking FUS aggregates (Fig. 5a, c, d). Immunostainings for EWSR1 and TAF15 also showed reduced nuclear levels in patient α-MNs [suppl. Figure 6 (Online Resource 2]). Interestingly, we also observed rare co-localization of hnRNPA1, EWSR1, and TAF15, but not hnRNPA2B1, with FUS aggregates in both α-MNs and cortical neurons [Fig. 5b as well as suppl. Figure 6c, d (Online Resource 2)]. Hence, FUS aggregation in ALS patient MNs affects other ALS-associated RBPs.

### Knockdown of ALS-associated RBPs reduces cell viability

**Fig. 6:**
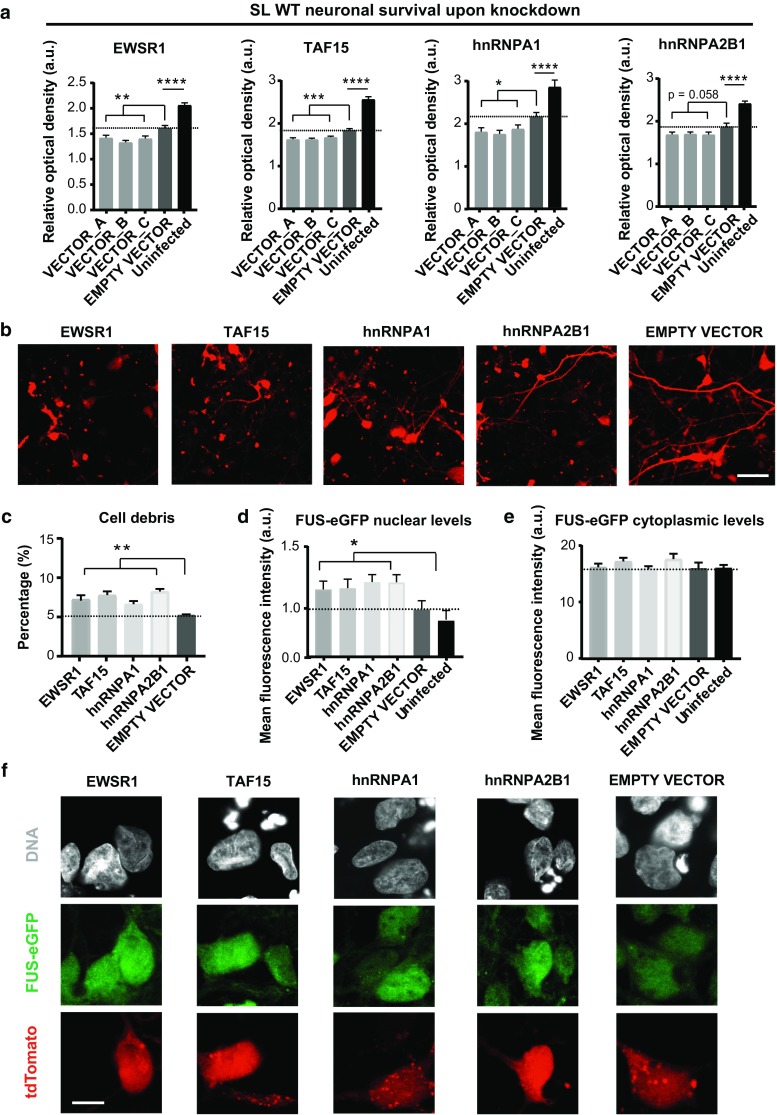
Reduction in RBP levels synergistically contributes to neurodegeneration. **a** Cell viability assay performed on neurons infected with either empty or knockdown vectors shows reduced viability following knockdown of the indicated RBP. A, B and C refer to three individual shRNA vectors, each targeting the indicated RBP. *n* = 3. Error bars indicate SEM. *, **, ***, and **** Correspond to *p* < 0.05, 0.01, 0.001, and 0.0001, respectively. **b** For each knockdown, a representative confocal micrograph of the culture is shown. Infected cells are identified by tdTomato expression. Scale bar = 50 µm. **c** Quantification of cell debris abundance following neuronal death induced by RBP knockdown. Results from the three individual vectors for each knocked-down protein were combined and compared to the empty vector. *n* = 3. **d**, **e** Mean fluorescence intensity of **d** nuclear and **e** cytoplasmic FUS-eGFP in iPSC-derived neurons after RBP knockdown. **f** Representative confocal micrographs. Scale bar 10 µm. *n* = 3. Error bars indicate SEM. * and ** Correspond to *p* < 0.05 and 0.01, respectively

Next, we asked whether reduced levels of ALS-associated RBPs contribute to neurodegeneration. To test this, we generated lentivectors carrying small hairpin RNA (shRNA) sequences targeting EWSR1, TAF15, hnRNPA1 and hnRNPA2B1 for gene knockdown [suppl. Figure 7 (Online Resource 2), suppl. Figure 8a (Online Resource 2), as well as suppl. Tables 5 and 6 (Online Resources 7 and 8)]. We first determined knockdown efficiency using HEK293T cells [suppl. Figure 8b (Online Resource 2)], and subsequently optimized infection conditions in SL WT FUS-eGFP neurons [suppl. Figure 9 (Online Resource 2)]. To evaluate neurodegeneration mediated by RBP knockdown, we lysed transduced neuronal cultures and probed the supernatant for LDH activity as a measurement of cell viability. Importantly, all RBP knockdowns caused increased neurodegeneration compared to controls (Fig. 6a). In line with this finding, knockdown of each EWSR1, TAF15, hnRNPA1, and hnRNPA2B1, caused iPSC-derived neurons to develop a strongly impaired morphology, as well as higher amounts of cell debris compared to controls (Fig. 6b, c). Of note, although hnRNPA1 knockdown caused a reduction in hnRNPA1 levels of only 10%, neuronal viability was considerably reduced, underlying the importance of RBP homeostasis in neurons, where even small changes in the abundance of these proteins can strongly impact on cell physiology.

We also tested the effects of reduced ALS-associated RBPs on FUS by quantifying FUS-eGFP fluorescence intensity in infected versus uninfected cells. We consistently detected increased SL WT FUS-eGFP nuclear signal upon knockdown of each of the tested RBPs (Fig. 6d, f). Simultaneously, we observed a slight but not significant increase in cytoplasmic FUS-eGFP, which is in agreement with the FUS-eGFP protein having a WT NLS. These results further validated the interdependence between FUS and the investigated ALS-associated RBPs (Fig. 6e).

### Inducing autophagy reduces accumulated P525L FUS and restores RBP homeostasis

**Fig. 7:**
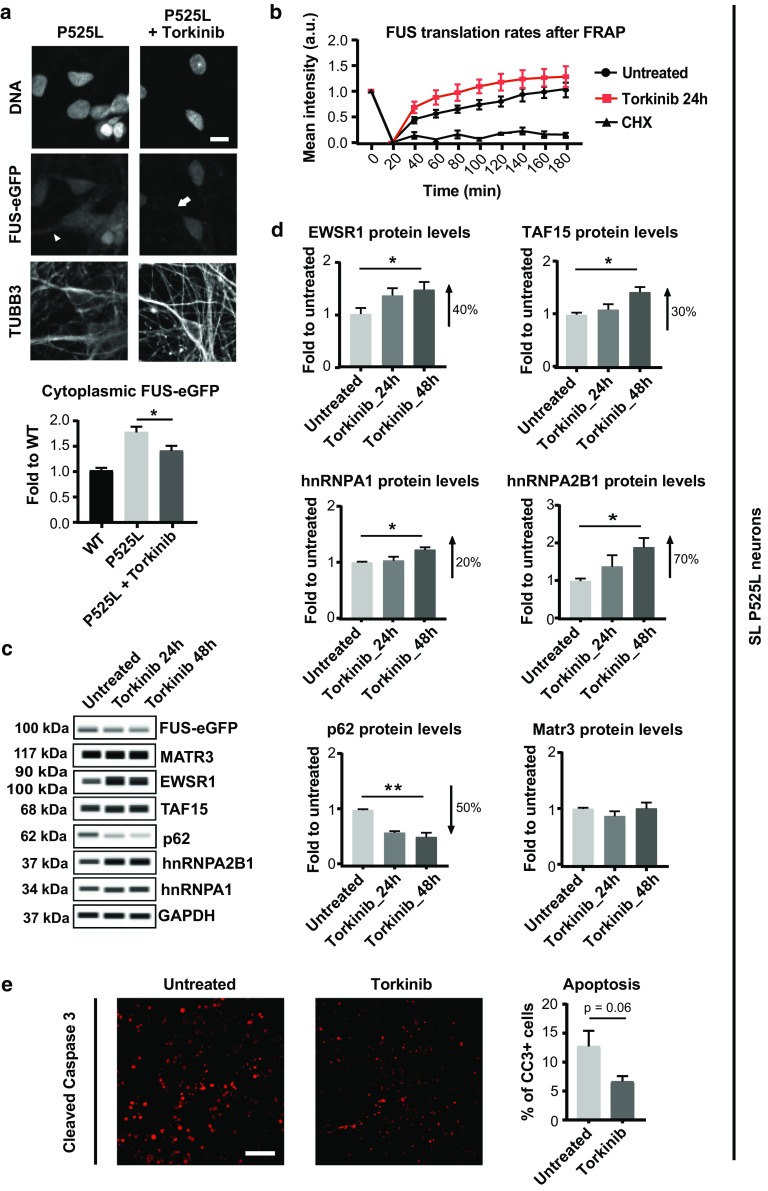
Autophagic clearance of aberrantly accumulated cytoplasmic FUS restores protein homeostasis and ameliorates survival of SL P525L iPSC-derived neurons. **a** Confocal micrographs showing FUS-eGFP distribution before and after Torkinib treatment (above). Arrowhead indicates FUS-eGFP cytoplasmic accumulation in untreated neurites; arrow shows reduced FUS-eGFP cytoplasmic signal following torkinib treatment. Quantification of cytoplasmic FUS-eGFP signal intensity in acquired images (below) confirms clearance of mislocalized FUS-eGFP protein. Scale bar = 10 µm. **b** FRAP analysis performed on untreated versus torkinib-treated neurons shows comparable dynamics of FUS-eGFP recovery. *n* = 3. Error bars indicate SEM. CHX = cycloheximide. **c** WES capillary electrophoresis and **d** corresponding quantification of the indicated proteins in P525L SL neurons before and after torkinib treatment. Autophagy stimulation restores physiological levels. *n* = 4. Error bars indicate SEM. * and ** Correspond to *p* < 0.05 and 0.01, respectively. **e** 6 h of torkinib reduces apoptotic cell death identified by cleaved Caspase 3 staining. Scale bar = 50 µm

We described that ALS patient MNs show heterogenous levels of FUS mislocalization, and we used iPSC-derived spinal neurons to model this heterogeneity. We showed that inhibition of autophagy increases the accumulation of cytoplasmic FUS, and we demonstrated that FUS accumulation is linked to a number of pathological phenotypes involving other ALS-associated RBPs. This led us to speculate that stimulating autophagy would facilitate the clearance of mislocalized FUS and restore protein homeostasis. To this end, we treated our neuronal cultures with the mTOR inhibitor torkinib. Because torkinib inhibits mTOR directly by preventing ATP binding, it induces autophagy in neurons more potently than rapamycin [(suppl. Figure 4f, g (Online Resource 2)], which uses a more indirect mechanism involving FKBP12 [18]. Quantification of FUS-eGFP signal fluorescence intensity demonstrated that torkinib treatment significantly reduced cytoplasmic FUS-eGFP levels compared to controls (Fig. 7a). Consistent with the reduction in cytoplasmic FUS-eGFP, we found that P525L neurons treated with torkinib displayed decreased FUS-eGFP recruitment to arsenite-induced cytoplasmic SGs compared to controls [suppl. Figure 10a (Online Resource 2)]. In addition, SL P525L neurons are characterized by an increased FUS aggregation propensity relative to WT, and torkinib induced a trend of amelioration [suppl. Figure 10b (Online Resource 2)]. Using capillary electrophoresis, we observed a time-dependent increase in EWSR1, TAF15, hnRNPA1, and hnRNPA2B1 protein levels upon torkinib treatment (Fig. 7c, d). In addition, torkinib decreased p62 levels by about 50% compared to untreated controls (Fig. 7c, d). Finally, we report that torkinib moderately improved survival of neurons with SL P525L FUS-eGFP (Fig. 7e). Taken together, these data demonstrated that inducing autophagy using torkinib promotes protein homeostasis as well as neuronal survival.

Since mTOR inhibition can also suppress protein synthesis, it could be argued that the effects of torkinib on FUS-eGFP levels are due to suppression of translation rather than increased autophagy. To rule this out, we evaluated FUS-eGFP translation rates by measuring FUS-eGFP recovery after photobleaching of whole neuronal cell bodies. Results showed that torkinib did not slow down FUS-eGFP signal recovery, confirming that FUS translation rates are not altered by the treatment (Fig. 7b), which corroborates the beneficial role of torkinib in stimulating FUS clearance by autophagy.

Although torkinib restored protein homeostasis in spinal neuronal cultures, torkinib does not cross the blood–brain barrier, thus limiting its use as an oral ALS therapeutic (although intrathecal delivery might be possible). Nevertheless, mTOR is a clinically validated drug target, and brain penetrant inhibitors are being actively developed. One example is PQR309, which is currently in phase II clinical trials for cancer treatment [31]. Consistent with our results using torkinib, we found that PQR309 reduced the amount of P525L FUS-eGFP recruited to SGs in neurons treated with arsenite, indicating effective autophagic clearance of cytoplasmic FUS-eGFP [suppl. Figure 10a (Online Resource 2)]. In addition, PQR309 significantly diminished FUS aggregation measured by filter trap assay [(suppl. Figure 10b (Online Resource 2)] and restored homeostasis of ALS-associated RBPs as well as p62 in iPSC-derived neurons [suppl. Figure 10c (Online Resource 2)]. Therefore, compounds inducing autophagy, such as torkinib and PQR309, could be attractive for treating ALS.

### Autophagy stimulation decreases motor dysfunction in vivo

**Fig. 8:**
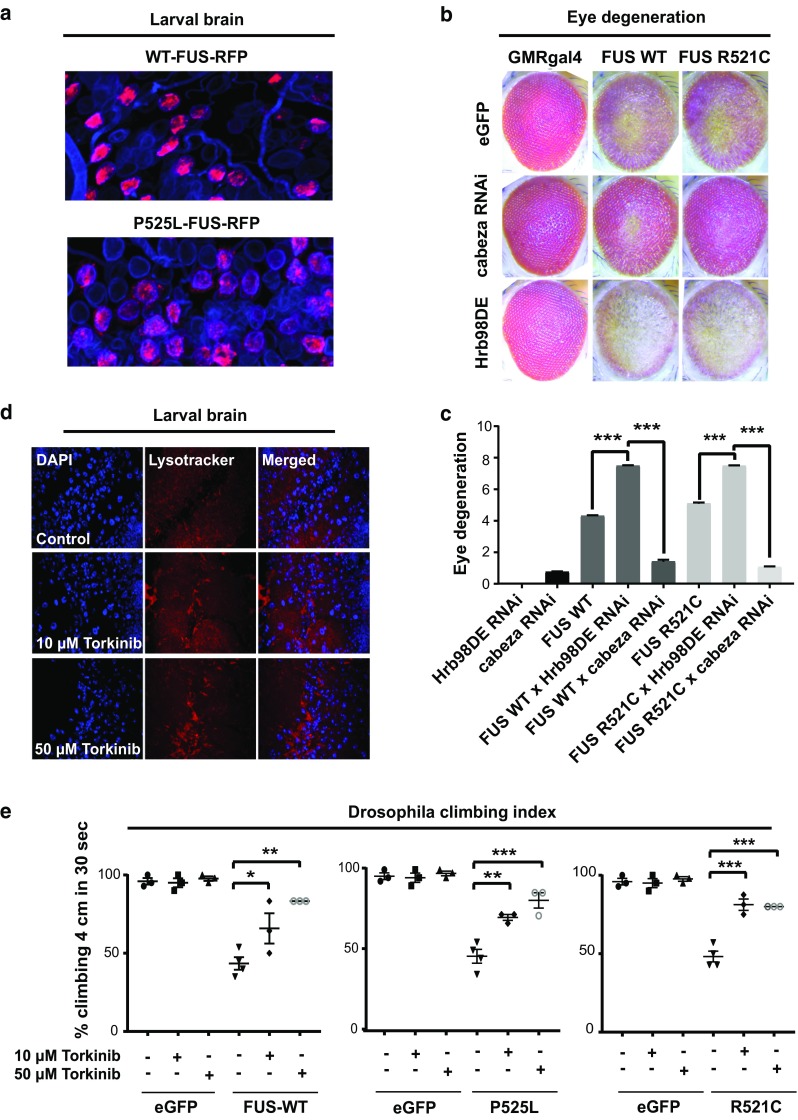
Drosophila models of FUS-ALS confirm the involvement of RBPs and are rescued by autophagy. **a** Localization of WT and P525L FUS-RFP in Drosophila larval brain. **b** WT and R521C FUS ectopic expression causes eye degeneration, which is rescued by cabeza RNAi and worsened by Hrb98DE RNAi. **c** Quantifications of eye degeneration. **d** Compared with or DMSO treated controls, Drosophila larvae fed with torkinib show autophagy induction in their brains as marked by increased Lysotracker Red staining. **e** Drosophila climbing assay showing improved fly climbing ability upon torkinib treatment at the indicated concentrations

Drosophila models have been instrumental in examining genetic and small molecule modifiers of neurodegenerative diseases, including FUS-ALS. Similar to ALS patients, P525L FUS-ALS flies exhibit heterogeneous cytoplasmic FUS levels (Fig. 8a). We aimed at demonstrating that knocking down RBPs exacerbates FUS pathogenesis in flies analogous to human neurons. Overexpression of either human WT FUS, which models a mutation in the 3′ untranslated region of FUS that upregulated FUS protein [25], or R521C in the fly eye resulted in degeneration (Fig. 8b, c). Consistent with our results using human neurons, RNAi against HRD98DE, which is the fly orthologue of hnRNPA1 and hnRNPA2B1, exacerbated the eye phenotype (Fig. 8b, c). Additionally, it was previously reported that knocking down Cabeza, which is the fly ortholog of human FET proteins FUS, EWSR1, and TAF15, causes neurodegenerative phenotypes in flies, including decreased adult viability, locomotor disabilities at both larval and adult stage, as well as reduced lifespan [30]. As expected, knocking down Cabeza rescued eye degeneration in flies overexpressing FUS (Fig. 8b, c).

Next, we tested if autophagy stimulation would ameliorate ALS phenotypes in vivo. To do this, Drosophila models of FUS-ALS were treated with torkinib. Selective overexpression of human WT FUS as well as two disease-causing mutations (R521C and P525L) in fly MNs caused severe motor dysfunction, as evident from reduced climbing ability (Fig. 8e). When we treated flies with two different concentrations (10 and 50 µm) of torkinib to induce autophagy (Fig. 8d), we found that treated animals showed a dose-dependent significant improvement in their climbing abilities (Fig. 8e), indicating that autophagy stimulation had suppressed FUS-induced motor dysfunction.

## Discussion

Multiple RBPs, including FUS, EWSR1, TAF15, hnRNPA1 and hnRNPA2B1 have been associated with ALS. Each of these RBPs has a low-complexity intrinsically disordered domain, which is prone to aggregation, and aggregates of these proteins are found in patients. One critical question is how healthy cells inhibit pathological phase transitions and maintain protein homeostasis. It was previously shown that FUS interactions with Transportin-1, as well as the high RNA concentrations in the nucleus prevent FUS aggregation in healthy cells and that ALS mutations disrupt these mechanisms [9, 17]. Here, we showed that mislocalization of FUS also disrupts its interactions with other ALS-associated RBPs that normally prevent FUS aberrant liquid-to-solid phase transition. Thus, although each of these proteins are individually prone to aggregation, mixing them together, as would be found in the nucleus of healthy neurons, buffers FUS against phase transition.

Here, we demonstrated that FUS-ALS is not solely restricted to FUS dysfunction and aggregation, but rather affects the homeostasis of a number of other ALS-associated RBPs. We showed that MNs in the spinal cord of FUS-ALS patients are heterogeneous in their degree of cytoplasmic FUS accumulation, supporting the idea of a progressive pathology. However, since FUS aggregates were described not to contain EWSR1 and TAF15 [20], it has long been thought that FUS-ALS pathology is primarily about FUS aggregation and does not affect other ALS-associated RBPs, implying that these ALS subtypes are essentially independent of each other. Instead, we showed that the presence of aggregation-prone FUS in the cytoplasm causes imbalances in RBP homeostasis, leading to a reduction in the nuclear levels of EWSR1, TAF15, hnRNPA1 and hnRNPA2B1, which exacerbates FUS pathogenesis and accelerates neurodegeneration. Using *post mortem* tissue from FUS-ALS patients, we observed that MNs with FUS aggregates also exhibit reduced overall levels of certain ALS-associated RBPs compared to MNs lacking FUS aggregation. Of note, reduced levels of hnRNPA1 and hnRNPA2B1 have been reported in cases of Alzheimer’s disease [2], suggesting a connection between RBP homeostasis and neuronal health in multiple disorders. Importantly, we demonstrated that this phenotype was not detectable in NPCs or undifferentiated iPSCs, highlighting the significance of studying ALS in human spinal neurons.

FUS-ALS accounts for approximately 35% of all patients developing the disease before 40 years of age, whereas mutations in other ALS genes, including *C9ORF72*, *SOD1* and *TDP43*, are much more common in older patients [26]. Additionally, some FUS mutations are associated with rapidly progressing juvenile forms emerging in the late teens and early twenties [26], including P525L. The unique aggressiveness of FUS-ALS might be linked to the involvement of so many RBPs, which synergistically contribute to neurodegeneration.

One interesting open question is the molecular mechanism by which the accumulation of cytoplasmic FUS leads to decreased levels of ALS-associated RBPs, including EWSR1, TAF15, hnRNPA1, and hnRNPA2B1. Transcriptional feedback may be a possibility, and one study described a transcriptional interdependency between FUS, EWSR1, TAF15 and hnRNPA1 [12]. However, this did not seem to be the case in our hands since RNA levels were unchanged. Another possibility is that RBP levels may be reduced by translational suppression, which was recently suggested by studies in a FUS mouse model [16]. Translational suppression could be linked to RNP granules, which act as sites for mRNA storage and/or degradation. This is particularly intriguing since FUS is a component of RNP granules, and mutant FUS causes RNP granules to aberrantly accumulate. However, explaining the specificity for EWSR1, TAF15, hnRNPA1 and hnRNPA2B1 is challenging, particularly when it has been found that FUS interacts with more than 5500 RNA targets, including the RBPs we investigated [13]. Interestingly, all of these RBPs, unlike TDP43 and Matrin3, have been experimentally validated as cargos for mammalian Transportin-1, which was described to play a role in P body formation and in the transport of specific cargoes to other RNP granules [29]. Thus, Transportin-1 might be a key player in this scenario. Finally, it is possible that RBP levels are reduced by protein degradation or excretion, for example by exosomes [10] or exophers [19]. Further experiments are needed to test these hypotheses.

Our results place impaired protein dynamics at the core of ALS pathogenesis, and we conclude that it is critical to restore appropriate levels of all ALS-associated RBPs to prevent neurodegeneration. A protein-specific gene therapy approach is unlikely to be effective since multiple ALS-associated RBPs were reduced by aberrantly accumulated mutant FUS, and the levels of these proteins are tightly regulated in neurons, with either too much or too little being toxic [5]. In our work, we demonstrated that stimulating autophagy could be an effective therapeutic to rescue protein homeostasis. We showed that spinal neurons with accumulated FUS are marked by increased p62 protein levels, suggesting defects in protein degradation, and, consistent with this, we observed aberrant lysosomal morphology. Importantly, stimulating autophagy with torkinib and PQR309 facilitated the clearance of cytoplasmic FUS and restored the levels of the investigated ALS-associated proteins. In line with these findings, small molecule-mediated induction of autophagy ameliorated MN function in Drosophila models of ALS. This implies that drugs modulating autophagy would likely be most effective when administered before protein aggregation initiates, which is presumably when patients are still asymptomatic. In this context, compounds inducing autophagy could be an effective strategy to protect neurons against the onset of FUS-ALS. Since defects in autophagy have been associated with both familial and sporadic ALS cases [14, 23], we propose that modulating autophagy could be an effective therapeutic for many ALS patients.

However, critical issues, including potential side effects and limitations, remain to be addressed before patients are treated with autophagy-enhancing drugs because inducing autophagy has occasionally been reported to exacerbate ALS in some models. For example, mTOR inhibition causes immunosuppression and was only beneficial in SOD1 G93A mice when lymphocytes were depleted [27, 32]. A possible strategy to overcome this would be to develop therapeutics capable of inducing autophagy in an mTOR-independent manner. Interestingly, it has been shown that autophagy plays different roles in MNs in SOD1 G93A mice depending on the disease stage [24]. Consistent with our findings, inhibiting autophagy accelerated muscle denervation and the onset of motoric phenotypes. However, later in the disease course, inhibiting autophagy unexpectedly prolonged survival. This suggests that, while autophagy stimulation may be a successful strategy for some ALS patients, any therapeutic approach must take into account multiple factors, including the genetic background, disease stage, and possibly other context-dependent influences.

## Electronic supplementary material

Below is the link to the electronic supplementary material.

Supplementary material 1 (DOCX 59 kb)

Supplementary material 2 (DOCX 61926 kb)

**Online Resource 3: Supplementary Table** **1. List of detected WT and P525L FUS protein interactors in LL iPSC-derived neurons.** The table summarizes the list of FUS interactors determined as protein enriched in either WT or P525L samples compared to the parental untargeted controls, which contained potential contaminants. Proteins are listed in descending order according to fold difference. (XLSX 53 kb)

**Online Resource 4: Supplementary Table** **2. GO biological process terms enriched in the list of common interactors between LL WT and P525L FUS protein.** The list was generated using FIDEA. GO terms are ranked in a descending order according to their significance. p-values were adjusted using the Benjamini–Hochberg method. (XLSX 53 kb)

**Online Resource 5: Supplementary Table** **3. Investigated RNA-binding proteins are not differentially expressed.** The list represents all the transcripts of interest detected by RNA profiling in SL P525L neurons versus SL WT neurons. Log2FoldChange shows no significant difference in gene expression. (XLSX 9 kb)

**Online Resource 6: Supplementary Table** **4. Summary of neuropathology cases and controls.** The table reports all relevant information for the human samples used in this study. (XLSX 12 kb)

**Online Resource 7: Supplementary Table** **5. Collection of primers used for Gibson assembly-mediated generation of knockdown constructs.** The table contains all primer sequences used in this work, flanked by a description of their purpose. The color code works as follows: purple = U6 promoter sequence overlap, grey = vector backbone overlap, orange = shRNA loop, red = poly-T. (EPS 2337 kb)

**Online Resource 8: Supplementary Table** **6. shRNA sequence information.** The table recapitulates the workflow followed for shRNA design. Open reading frames (ORFs) of the most abundant protein-coding transcript variants were selected for in silico design of RNAi molecules, which were ranked according to a score predicting knockdown probability. The utilized color code works as follows: orange = shRNA loop, red = poly-T. (EPS 1381 kb)
