# Supplementary material for: FUS pathology in ALS is linked to alterations in multiple ALS-associated proteins and rescued by drugs stimulating autophagy
Source: Acta Neuropathol. 2019 Apr 1;138(1):67–84. doi: 10.1007/s00401-019-01998-x (PMC6570784; doi:10.1007/s00401-019-01998-x)
Supplement: Supplementary file 1 — Supplementary material 1 (DOCX 59 kb) [file 401_2019_1998_MOESM1_ESM.docx]

FUS pathology in ALS is linked to alterations in multiple ALS-associated proteins and rescued by drugs stimulating autophagy

**Authors:** Lara Marrone^1^, Hannes C.A. Drexler^2^, Jie Wang^3^, Priyanka Tripathi^4^, Tania Distler^1^, Patrick Heisterkamp^1^, Eric Nathaniel Anderson^5,6^, Sukhleen Kour ^5,6^, Anastasia Moraiti^1^, Shovamayee Maharana^3^, Rajat Bhatnagar^7^, T. Grant Belgard^7†^, Vadreenath Tripathy^1^, Norman Kalmbach^8^, Zohreh Hosseinzadeh^1^, Valeria Crippa^9^, Masin Abo-Rady^1^, Florian Wegner^8^, Angelo Poletti^9^, Dirk Troost^10^, Eleonora Aronica^10^, Volker Busskamp^1^, Joachim Weis^4^, Udai Bhan Pandey^5,6,11^, Anthony A. Hyman^3^, Simon Alberti^3^, Anand Goswami^4^ and Jared Sterneckert^1*^

**Affiliations:**

^1^Center for Regenerative Therapies Dresden, Technische Universität Dresden, Fetscherstr. 105, 01307 Dresden, Germany.

^2^Max Planck Institute for Molecular Biomedicine, Bioanalytical Mass Spectrometry, Röntgenstr. 20, 48149 Münster, Germany.

^3^Max Planck Institute of Molecular Cell Biology and Genetics, Pfotenhauerstr. 108, 01307 Dresden, Germany.

^4^Institute of Neuropathology, RWTH Aachen University Hospital, Pauwelsstr. 30, 52074, Aachen, Germany.

^5^Department of Pediatrics, Division of Child Neurology, Children's Hospital of Pittsburgh, University of Pittsburgh School of Medicine, Pittsburgh, PA, United States.

^6^Department of Human Genetics, University of Pittsburgh Graduate School of Public Health, Pittsburgh, PA, United States.

^7^Verge Genomics, San Francisco, CA, United States.

^8^Department of Neurology, Hannover Medical School, Carl-Neuberg-Str. 1, 30625 Hannover, Germany

^9^Dipartimento di Scienze Farmacologiche e Biomolecolari, Centre of Excellence on Neurodegenerative Diseases Università degli studi di Milano, Milan 20133, Italy.

^10^Amsterdam UMC, University of Amsterdam, department of (Neuro)Pathology, Amsterdam Neuroscience, Amsterdam, the Netherlands.

^11^Department of Neurology, University of Pittsburgh School of Medicine, Pittsburgh, PA, United States.

*To whom correspondence should be addressed. Email: jared.sterneckert@tu-dresden.de, Phone: +49 351 458 82103, Fax: +49 351 458 82119.

†Current address: The Bioinformatics CRO, Niceville, FL, United States.

**Acta Neuropathologica Online Resource 1: Supplementary Materials and Methods**

## Ethical Approval

All procedures involving human participants were performed in accordance with the ethical standards of the institutional and/or national research committee as well as with the 1964 Helsinki declaration and its later amendments or comparable ethical standards.

## Cell culture and treatments

Cell lines used in this study were previously obtained and characterized [6]. Briefly, iPSC-derived neural progenitors (NPCs) were cultured in N2B27 medium - Neurobasal medium 1:1 DMEM F12 medium supplemented with N2 and B27 (all ThermoFisherScientific), as well as penicillin/streptomycin/glutamine (MerckMillipore) - including 3 µM CHIR 99022 (AxonMedChem), 200 µM ascorbic acid (Sigma) and 0.5 µM PMA (Cayman). NPCs were typically split 1∶10 every 6 days using Accutase (Sigma) and distributed on Matrigel (Corning)-coated plates. To trigger neuronal differentiation, NPC expansion medium was replaced by patterning medium. This consisted of N2B27 medium supplemented with 200 µM ascorbic acid, 1 µM retinoic acid (Sigma), 0.5 µM CHIR, 10 ng/ml BDNF, and 10 ng/ml GDNF (both Peprotech). After 6 days, cells were fed with maturation medium (N2B27 with 5 ng/ml Activin A (eBioscience - only for the first 2 days), 1 ng/ml TGFβ3 (Peprotech), 200 µM ascorbic acid, 20 ng/ml BDNF, 20 ng/ml GDNF, 500 µM dbcAMP (Sigma). Two days later, neurons in their early maturation stage were re-seeded in the desired plate format, and further kept in culture for at least two weeks prior to analysis. For live-cell imaging, cells were re-plated on 35 mm glass-bottom dishes previously coated with laminin at a density of 300,000 cells/dish. For laminin coating, dishes were initially treated with poly-L-ornithine solution (PLO, Sigma) in PBS overnight at 37^o^ C. Subsequently, laminin (Biolamina) was dissolved in PBS and pipetted onto the PLO-coated surface. Sodium arsenite treatment (0.5mM) was performed for 1h. All other treatments were accomplished over 24h or 48h. Working concentrations are listed as follows: Torkinib (10 µM), PQR309 (10 µM), Adox (100 µM), MG132 (5 µM), 3-MA (2.5 mM). Compounds were dissolved in plain N2B27 medium. HEK293T cells were cultured in 10 % FBS in DMEM High Glucose supplemented with Penicillin/Streptomycin/Glutamine (Thermo Fisher Scientific).

## Immunostaining

Cells were fixed with 4 % PFA (EM Sciences) in PBS for 20 min. Permeabilization and blocking were done in a single step by applying 0.1 % Triton (Roth), 10 % FCS (GE Healthcare) and 1 % BSA (Roth) in PBS for 45 min. Incubation with primary antibodies was performed over night at 4 °C. Secondary antibodies were added for one hour at room temperature. All antibodies were diluted in 0.1 % BSA in PBS. Eventually, cells were incubated for 5 min with Hoechst (Thermo). In between each step, cells were washed 0.1 % BSA in PBS and finally left in PBS for imaging. The following primary antibodies were used: rabbit anti-MAP2 (sc-20172, Santa Cruz) 1:1000, mouse anti-TUBB3 (BLD-801202, Biolegend) 1:1000, rabbit anti-cleaved caspase 3 (9661S, Cell Signaling) 1:400, mouse anti-p62 (ab56416, Abcam) 1:500. Secondary antibodies included: donkey anti-rabbit AlexaFluor 568 (A-10042, Thermo) and donkey anti-mouse AlexaFluor 647 (A-31571, Thermo), all 1:1000. Lysosomes were labelled using Lysotracker Red DND-99 (Thermo) by diluting the fluorescent dye 1:1000 in N2B27 medium and incubating the cells for 1h before fixation.

**Proximity ligation assay**

Proximity ligation was performed using the Duolink In Situ Red Starter Kit/MouseRabbit (Sigma-Aldrich) as per manufacturer’s instructions. Briefly, 70,000 neurons were fixed as above, and permeabilized with 0.5% Triton-X 100 (Roth) in PBS for 10 min, followed by 3x 5 min washes with 0.05% Tween-20 (Applichem). Blocking, antibody incubation, probe incubation, ligation and amplification were performed according to the manufacturer’s protocol using the provided reagents. Primary antibodies included: mouse anti-FUS 1:500 (AMAB90549), rabbit anti-FUS 1:400 (ab84078), mouse anti-hnRBPA1 1:200 (NB100-672) and mouse anti-hnRBPA2B1 1:200 (sc-32316). Image acquisition was performed with a Zeiss confocal laser scanning microscope using a 40x magnification.

**Confocal microscopy, fluorescence recovery after photobleaching (FRAP) and image analysis**

Images of fixed cells were acquired using a confocal laser scanning microscope 700 from Zeiss. Quantification of cytoplasmic FUS-eGFP signal was performed using Cell Profiler. For this, a workflow was developed to first recognize cell nuclei via Hoechst and neurites via MAP2 staining. The FUS-eGFP mean integrated fluorescence intensity within neurites - excluding nuclear signal - was then calculated. Nuclear FUS-eGFP signal intensity was obtained by manually drawing regions of interest (ROIs) using Fiji, followed by measurement of the mean fluorescence intensity signal within the ROI. Alternatively, whole confocal micrographs were processed by first converting them to black and white images according to a specific threshold that would enable to clearly distinguish all nuclei (“Image” -> “Adjust” -> “Threshold”). Subsequently, mean integrated fluorescence intensity of FUS-eGFP was obtained by running the “analyze particle” command for objects of a sufficiently extended size to include each nucleus. For stress granule (SG) analysis, confocal micrographs were first corrected by reducing the noise via the “subtract background” command in the “Process window”. Subsequently, they were converted to black and white images as described above, with a threshold that would highlight SGs. SGs were defined as objects with 0.1-1.0 circularity and 1-3 µM size. Cleaved caspase 3 (CC3) stainings were analyzed via Cell Profiler by detection of dual Hoechst- and CC3-positive cells. tdTomato-positive neurons were either counted manually using the Fiji tool “Cell counter”, or in an automated fashion using the IncuCyte Cell analysis system (Sartorius) to detect the area occupied by red fluorescent signal per well. Cell debris count was also performed using IncuCyte software by calculating the percentage of red fluorescent particles smaller than a threshold empirically determined to only encompass cell fragments. For FRAP analysis, whole neuronal cell bodies were photobleached to measure the translation rate of FUS-GFP by assessing the GFP fluorescence recovery. Cells treated with 100 µM cycloheximde for 1.5 h were used as a negative control. Neurons were analyzed during early maturation. Experiments were performed at a pixel resolution of x = 100 nm and y = 100 nm, using a 60x oil immersion objective mounted on an Olympus IX71/IX81 inverted confocal microscope with Andor spinning disc and Andor iXon EMCCD camera. Multiple bleach points within each bleached neuron were selected to ensure whole cell body coverage. 60-70 % of maximum laser power of a 488-nm laser (3.5 mW) was used for 3 rounds photobleaching, each of 50 ns residence time. Five images were acquired before bleaching, and an average of these was used to calculate the pre-bleach intensity. After bleaching, images were captured for 4h, 20 min interval, to record the signal recovery associated with newly translated GFP-tagged FUS.

**Capillary electrophoresis, western blotting, filter retardation assay and immunoprecipitation**

For capillary electrophoresis and immunoblotting, cells were lysed on ice using RIPA buffer supplemented with protease inhibitors (Santa Cruz Biotechnology). Protein concentrations were determined via BCA assay (Pierce™ BCA Protein Assay Kit, #23225, Thermo Fisher Scientific) following manufacturer’s instructions. Cell lysates were analyzed by capillary electrophoresis using the 12-230 Separation Module associated with the Protein Simple WES™ device. Here, all reagents except primary antibodies were provided with the manufacturer’s kit (ProteinSimple). Samples were loaded at a concentration of 0.4 µg/µl. Primary antibodies included: rabbit anti-GFP (sc-8334 Santa Cruz) 1:50, mouse anti hnRNPA1 (NB100-672, Novusbio) 1:1000, mouse anti-hnRNPA2B1 (sc-32316, Santa Cruz) 1:100, mouse anti alpha Tubulin (T9026, Sigma) 1:500, rabbit anti-EWSR1 (NB200-182, Novusbio) 1:50, rabbit anti-TAF15 (NB100-567, Novusbio) 1:100, mouse anti-p62 (ab56416, Abcam) 1:50, rabbit anti-Matrin3 (A300-591A, Bethyl) 1:250, rabbit anti-TDP43 (12892-1-AP, Proteintech) 1:50, rabbit anti-GAPDH (2118S, NEB) 1:500. For western blot, 20 µg of the protein lysate were mixed with 5x Laemmli buffer and loaded on 10 % or 12 % SDS PAGE separation gels after incubation at 95 °C for 5 min. Blotting was performed on a nitrocellulose membrane overnight. The membrane was then blocked for 1 h in 5 % milk in TBS-T 1X, followed by incubation with the primary antibody overnight at 4^o^C. After washing, the membrane was incubated with HRP-coupled secondary antibodies and eventually developed with ECL solution (GE Healthcare). Signal was detected with an image analyzer ImageQuant LAS 4000 (GE Healthcare). The following primary antibodies were used: rabbit anti-GFP 1:400, rabbit anti-GAPDH 1:5000, mouse anti-hnRNPA2B1 1:100, rabbit anti-EWSR1 1:5000, rabbit anti-TAF15 1:5000, mouse anti-p62 1:1000, rabbit anti-Matrin3 1:5000, rabbit anti-TDP43 1:1000, rabbit anti-LC3 (MBL-PM036, MBL) 1:1000. Horseradish peroxidase-conjugated anti-mouse and anti-rabbit antibody (711-035-152/ 711-035-150, Dianova) were used as secondary antibodies at a dilution of 1:10 000. Images were analyzed manually with Fiji and plotted using GraphPad Prism 7. For filter retardation assay, 20 µg of the total proteins were filtered through a 0.2-μm cellulose acetate membrane (Whatman, 100404180). Membranes were probed as described for western blotting using a rabbit anti-GFP antibody. For immunoprecipitation of FUS-eGFP fusion protein, the GFP-Trap® Kit from Chromotek was used. Briefly, neurons from a fully confluent 10-cm dish were lysed in the provided lysis buffer supplemented with Complete protease inhibitor cocktail (Roche). Equilibrated GFP-Trap® beads were added to the diluted lysate and incubated for 1h at 4^o^ C. After washing, bound proteins were eluted with 0.2 M glycine pH 2.5 followed by neutralization with 1M Tris base pH 10.4. Immunoprecipitated proteins were then analyzed either via immunoblotting or LC-MS.

**Mass spectrometric sample preparation and analysis**

Eluted samples were first acetone-precipitated over night to remove salts and detergent remnants. Protein pellets were dissolved in 8M urea, 10 mM HEPES, pH 7.5, reduced with 10mM DTT for 1 hr at 37° C prior to alkylation with 55 mM JAA for 30 min at RT in the dark followed by predigestion with 0.5 µg endopeptidase LysC ( Wako Chemicals, Neuss) at 37° C for 3-4 hours. After diluting the urea concentration to 2M, 0.5 µg trypsin was added (Promega) and the digest continued at 37° C overnight. The digest was stopped by TFA addition to a final concentration of 0.1 % and desalted using Stage tips as previously described [8]. All samples were prepared as biological replicates (n=3) for label free quantification and stored at 4° C until subjected to LC-MSMS analysis. Peptides were analyzed by LC MS/MS on a Q Exative HF mass spectrometer (ThermoFisher Scientific), equipped with an Easy nano-LC 1200 system and a EasyFlex electrospray source (ThermoFisher Scientific). Briefly, peptides were online-separated by reversed-phase chromatography on fused silica capillary chromatography columns (25 cm length, ID 75 µm; NanoSeparations) that were packed in-house with Reprosil pur C18 material (3 µm; Dr. Maisch, Ammerbuch, Germany), using a gradient from 2 to 40 % of buffer B (80 % acetonitril, 0.1 % formic acid) in 220 min and from 40 – 60 % B in 20 min at a flow rate of 250 nl/min. After additional 12 min at 90 % B the column was re-equilibrated at starting conditions. The mass spectrometer was operated in data-dependent mode (source voltage 2.1 kV) automatically switching between a survey scan (mass range m/z = 300-1750, resolution R = 60 K; AGC target value 3e6, maximum IT 100 ms) and MS/MS acquisition of the 17 most intense peaks by higher-energy collisional dissociation (resolution 15K; AGC target 1e5; max IT 50 ms; isolation width m/z = 1.6; normalized collision energy 27 %; dynamic exclusion enabled for 30.0 s; double charge and higher charges were allowed). Raw data were processed by MaxQuant software (1.6.0.13) involving the built-in Andromeda search engine [2]. The search was performed against the human uniprotKB database UP000005640_9606.fasta (version from 12/2015) supplemented with common contaminants with a 1 % FDR at the peptide and protein level. Parameters defined for the search were: Trypsin as digesting enzyme, allowing two missed cleavages; a minimum length of 7 amino acids; carbamidomethylation at cysteine residues as fixed modification, oxidation at methionine and protein N-terminal acetylation. Maximum allowed mass deviation was 20 ppm for MS and 0.5 Da for MS/MS scans. Peptide identiﬁcations by MS/MS were allowed to be transferred between runs after retention time alignment to minimize the number of missing values for the subsequent label free quantiﬁcation process, performed with the MaxLFQ algorithm using a minimum ratio count of 1. Mass spectrometry data were deposited to the PRIDE repository via ProteomeXchange under the following identification number: PXD010966 (<http://proteomecentral.proteomexchange.org>) [9]. Perseus software (version 1.6.0.7) was used for statistical and bioinformatics analysis. Proteins that were identiﬁed in the decoy reverse database or only by a site modiﬁcation, as well as common lab contaminants were excluded from further data analysis. In additon, a minimum of 2 peptides was required for protein identification, one of which had to be unique to the proteingroup. Log2 transformed LFQ values were further ﬁltered to ensure that expression values were present in at least 2 biological replicates of at least one experimental group. Missing values were substituted by imputation (down shift = 1.8 and width = 0.3). For binary comparisons of experimental groups Student´s T-test was used with a p-value of 0.05 as truncation value. Significantly altered proteins were normalized by z-scoring and analyzed by hierarchial clustering using Euclidian distance for column and row clustering.

**Protein purification and suppressing fiber assay**

FUS P525L was first expressed in SF9 insect cells and harvested 72h post-infection. Cells were collected by centrifugation for 5 min at 2,000 rpm. Pellets were re-suspended in lysis buffer (50 mM Tris-HCl pH 7.4, 1 M KCl, 5 % Glycerol and 10 mM Imidazole). Protease inhibitors (Calbiochem, 1 mM PMSF, 100 μM AEBSF, 0.08 μM Aprotinin, 5 μM Bestatin, 1.5 μM E-64, 2 μM Leupeptin and 1 μM Pepstatin A) were added and cells were lysed by sonication. The crude lysate was clarified by centrifugation for 15 min at 13,000 rpm. After centrifugation, the supernatant was loaded onto amylose resin (NEB) columns. Protein-bound beads were further washed with 2 column volumes (CV, 1 CV = 20 ml) of lysis buffer and proteins were eluted with elution buffer (50 mM Tris-HCl pH 7.4, 500 mM KCl, 5 % Glycerol and 10 mM Maltose). For His-MBP tag cleavage, 3C pre-scission protease was added to the eluate at a 1:50 ratio. The mixture was incubated at RT for 3h and concentrated. The protein was further purified over the gel filtration chromatography (Superdex-200; GE Healthcare) equilibrated with storage buffer (50 mM Tris-HCl, 500 mM KCl, 1 mM DTT and 5 % Glycerol). The cleaved His-MBP was separated from FUS P525L via gel filtration chromatography. Peak fractions were pooled and aliquoted in PCR tubes, flash-frozen in liquid nitrogen and stored at -80^o^ C. To assess the effect of distinct prion-like proteins on fiber formation, the indicated amount of prion-like proteins was mixed with 4 μM FUS P525L in phase separation buffer, containing 7.5 mM Tris-HCl 7.4, 75 mM KCl and 0.75 % Glycerol. Phase separation was induced by reduced salt concentrations in the mixture. The mixture containing phase separated proteins was added into the 384 well non-binding microplates. To induce fiber formation, the plate was shaken at 800 rpm on a benchtop shaker at RT. Imaging and FRAP recordings were performed at specified time points. Images were acquired using an IX71/IX81 inverted Spinning Disc Microscopes with an Andor Neo sCMOS/Andor Clara CCD camera and a 60x water-immersion objective. Images were analyzed with FIJI (http://fiji.sc/), origin 7 (OriginLab) and Sigmaplot (Systat Software Inc.).

**RNA sequencing**

1 million neurons per genotype were harvested in duplicate from 2 separate differentiation rounds, for a total of 4 samples per line. Cells were lysed in RLT plus buffer (Quiagen), and RNA was extracted using the RNeasy Plus Kit (Quiagen) according to manufacturer’s instructions. The quality of RNA sequencing reads from fastq files was assessed by the open source software FastQC. The average quality score for each sample indicated high base call accuracy when >30. The GC-content of reads across samples was evaluated for exhibiting an approximate normal distribution. To obtain transcript-level quantifications, reads were pseudo-aligned to the reference genome GRCh38 from ensemble.org with the software package Salmon. After excluding genes with low read counts (below 5 counts per million), read counts were normalized using the regularized log transformation method. Differentially expressed genes between genotypes were determined via the R software package DESeq2 with standard settings. The same method was applied to determine differentially expressed transcripts.

**Lentivector design, generation and transfection**

Three different shRNA sequences for each EWSR1, TAF15, hnRNPA1 and hnRNPA2B1 were designed using the online tool BLOCK-iT™ (Thermofischer). Primer synthesis was outsourced to Metabion. Double strand shRNA fragments were obtained by PCR-amplification of obtained primers (listed in Table 3 as F4-R4) using the following conditions in the presence of Phusion HF DNA-Polymerase (NEB): initial denaturation for 1 min at 98^o^ C, followed by 35 cycles consisting of denaturation at 98^o^ C for 30 sec, annealing at 60^o^ C for 15 sec, extension at 72^o^ C for 15. The reaction was terminated with an elongation step at 72^o^ C for 1 min. The U6 promoter was PCR amplified from a pre-existing vector using the same conditions. The lentiviral vector backbone was built in our lab by assembling two fragments derived from the vectors p-CAG-BFP-pCAG-tdTomato and p-INDUCER20-Tau, respectively. In particular, p-CAG-BFP-pCAG-tdTomato was digested with EcoRV and HpaI (both Promega), while p-INDUCER20-Tau was digested with BamHI and NcoI (both promega). Fragments containing the tdTomato cassette and lentiviral elements, respectively, were extracted from a 0.8 % agarose gel using the GeneJET Gel Extraction Kit (ThermoFischer Scientific). These fragments were joined by Gibson assembly (E2611, NEB) in the presence of the linking sequences F1-R1 and F2-R2 (Table 3), the latter containing a unique KpnI restriction site for vector linearization. This vector was referred to as “empty vector”. After evaluation of vector correctness, the backbone was linearized via KpnI digestion and the shRNA sequences F4-R4 were cloned along with the U6 promoter sequence into the plasmid backbone via a second round of Gibson Assembly according to manufacturer’s instructions. Successful insertion of the U6 promoter sequence was assessed via restriction digestion using KpnI, PvuI and NdeI (all Promega). Sanger sequencing by Eurofins Genomics confirmed correct insertion of the shRNA fragments. For plasmid amplification, home-made Stbl3 competent *E. coli* were transformed using a heat-shock step at 42^o^ C for 45 sec, followed by recovery. For competent cell preparation, Stbl3 bacteria were incubated on ice for 15 min after reaching their exponential expansion phase. Cells were centrifuged for 15 min, 800 xg at 4^o^ C. The pellet was resuspended in 100 mM CaCl_2_ solution and further incubated on ice for additional 30 min, followed by another centrifugation step. Bacteria were finally resuspended in 100 mM CaCl_2_/15 % Glycerol solution and aliquoted. Plasmid DNA was amplified at either a small or a large scale, and purified with a ZR Plasmid Miniprep Kit (Z#D4015, Zymo Research) or a NucleoBond Xtra Midi/Maxi kit (#740414 Macherey-Nagel), respectively. The procedure was performed according to manufacturer’s instructions. Amplified plasmids included the Gibson Assembly-generated lentivectors, as well as the plasmids required for viral vector production, i.e. pCD/NL-BH and pczVSV-G wt (kindly donated by Prof. Dr. Calegari, CRTD, Dresden). For transient plasmid expression, HEK cells were transfected in the presence of polyethylenimine (PEI) at a final concentration of 1µg/ml.

## Lentiviral particle production and transduction

A transient triple transfection of HEK293T cells was used to produce HIV‑1‑derived replication incompetent lentiviral particles, which were pseudotyped with the envelope protein VSV-G. For transfection, HEK293T cells were plated onto 10 cm dishes at a confluence of about 30-40 %. Before transfection, cells were fed with 4 ml culture medium. PEI was mixed with an equal volume of DMEM high glucose; for each 10-cm dish, 1 ml in total. Separately, a transfer vector/ pCD/NL-BH (GAG-POL)/ pczVSV-G mix was assembled following a 4:2:1 ratio. In particular, for each 10-cm dish, 8.4 µg transfer vector, 4.2 µg pCD/NL-BH (GAG-POL) plasmid and 2.1 µg pczVSV-G plasmid were diluted in 1 ml DMEM high glucose and mixed with the PEI/DMEM solution. The mixture was incubated at 37^o^ C for 15 min and subsequently added dropwise to the HEK293T cell culture. On the first day after transfection, medium was changed. On days 2 and 3, the supernatant containing budding lentiviral particles was harvested and stored at 4 °C. This was then centrifuged at 500 g for 5 min, followed by filtration through a 0.45 µm filter, and subsequent ultracentrifugation 22,500 xg for 3.5 h at 4^o^ C. The invisible pellet was resuspended in 50 µl PBS per initial plate, and the concentrated virus was stored at ‑80 °C after aliquoting. For infection, 90.000 neurons/well were seeded on 96-well plates, and infected after 10 days of maturation with the produced lentiviral vectors at a 1:50 dilution (following titration) in neuronal medium supplemented with protamine sulfate (Sigma-Aldrich) to a final dilution of 10 µg/ml. Cells were maintained in culture for up to 9 days after transduction, and the medium was exchanged on a daily basis until analysis.

**LDH cytotoxicity assay**

The LDH cytotoxicity assay was performed in an unconventional way compared to standard protocols. Briefly, cells were lysed 9 days after infection via incubation in 2 % Triton X-100 (Sigma Aldrich) in maturation medium for 24 h. Lysed cells released LDH in the supernatant, whose concentration was determined using an LDH cytotoxicity detection kit (TAKARA, #MK401) combined with colorimetric signal quantification via a Synergy Neo plate reader (BioTek).

**Drosophila experiments**

All Drosophila stocks were maintained on standard cornmeal at 25 °C in light/dark controlled incubator. The w1118, UAS-eGFP, and D42-GAL4 were obtained from the Bloomington stock center. The UAS-FUS WT, UAS-FUS P525L, and UAS-FUS R521C were previously described [1]. Climbing assay was performed as previously described [1]. Briefly, flies expressing FUS, eGFP or w1118 were grown in the presence or absence of either PP 242/Torkinib or PQR309 (both MedChemExpress) at a concentration of 10µM or 50µM, then anesthetized, placed into vails and allowed to acclimatize for 15 mins in new vails. Each genotype of flies was knocked three times on the base of the bench and a video camera was used to record the flies climbing up the wall of the vials. The percentage of flies climbing 4cm in 30 second was quantified, and the mean of each group was calculated and analyzed using Graphpad Prism 6 software. Statistical analysis was carried out using either T-test or one-way ANOVAs with Tukey’s or Dunnet’s multiple comparisons test. P < 0.05 was considered as statistically significant. For autophagy investigation, fly larvae were treated with torkinib (10µM and 50µM) or DMSO. Dissections were performed as previously described [3]. Briefly, wondering 3rd instar larvae raised with torkinib or DMSO were dissected in PBS. Brains were placed in PBS supplemented with 50µM lysotracker Red (Thermo) for 3mins, followed by 3x washes and fixation in 4 % Paraformaldehyde. After three more washing steps in 0.1% PBS-T, slices were mounted on coverslips and imaged right away.

**Case selection, immunohistochemistry and immunofluorescence of patient samples**

Human post-mortem brain and spinal cord (lumbar, thoracic, cervical) samples fixed in buffered formalin (n = 6 age-matched controls, n = 6 FUS mutation (R521C) were obtained from the Department of (Neuro)Pathology, Academic Medical Center (AMC), University of Amsterdam, in compliance with the provisions of the local ethical commission (protocol: W11_073) and with the Declaration of Helsinki. Tissues were processed within 6–24 h after death. All ALS patients had suffered from clinical signs and symptoms of lower and upper MN disease. Age-matched control patients did not show any neuropathological anomalies. 3-4 µm paraffin sections were placed on poly-L-lysine coated slides and allowed to dry in an oven (37 ^o^C) overnight for immunohistochemistry as described in detail elsewhere [5]. Briefly, after deparaffinising the sections in xylene for 20 min, sections were rehydrated in 100 %, 95 % and 75 % ethanol for 5 minutes each followed by endogenous peroxidase quenching (H_2_O_2_ in deionized water) for 15 min. For antigen retrieval, sections were heated in citrate buffer, pH 6 (DAKO) for 15 min in a pressure cooker. After washing in PBS, sections were incubated with the primary antibody for 1 h, followed by incubation with an appropriate secondary antibody (IL immunologic, NL). After washing in PBS, sections were incubated with horseradish peroxidase (HRP) (DCS Innovative Diagnostic System) for 15 min. DAB reagent (DCS Innovative Diagnostic System DAB kit) was used to stain the sections which were then counter-stained with 6 % haematoxylin for 3 min. Sections were dehydrated in 70 %, 95 %, 100 % ethanol and in xylene for 5 min each, and were mounted in vitro-cloud mounting medium (R. Langenbrinck) for light microscopy. All procedures were performed at room temperature. Single and double immunofluorescence stainings were performed as previously described [4]. In brief, deparaffinised tissue sections were heated in citrate buffer, pH 6 (Dako) for 15 min in a pressure cooker. Sections were then blocked with 10 % ready to use blocking solution (Thermo Scientific) for 1 h at room temperature before incubating with primary antibody at 4 ^o^C overnight. After washing in TBS-T for 10 min, sections were incubated with Alexa conjugated secondary antibody (1: 500 dilutions in PBS) at room temperature for 2 h. Sections were then washed in TBS-T (2 x 10 minutes) and stained for 10 min with 0.1 % Sudan Black in 80 % ethanol to suppress endogenous lipofuscin auto-fluorescence. Finally, they were washed for 5 min in TBST and mounted with Vectashield (Vector lab) mounting medium containing DAPI. Images were taken using a Zeiss Axioplan microscope with an Axio Cam 506 colour camera for DAB staining and with a Zeiss LSM 700 laser scanning confocal microscope for immunofluorescence staining, respectively. Confocal images were analysed using adobe Photoshop CS5 and ZEN 2009 software.

**Electrophysiology**

Whole-cell patch-clamp recordings of differentiated neurons were performed as described previously [7] during week 7 of differentiation at RT using an inverted microscope (Zeiss). In brief, whole-cell currents were low-pass filtered and digitized at 2.9 kHz and 10 kHz using an EPC-10 amplifier (HEKA). PatchMaster and FitMaster software (HEKA) was used for recording and final analysis, respectively. Borosilicate glass pipettes (Science Products) were pulled to yield a resistance of 3-4 MΩ when filled with the internal solution (153 mM KCl, 1 mM MgCl_2_, 10 mM HEPES, 5 mM EGTA and 2 mM Mg-ATP, calibrated to pH 7.3 with KOH; 305 mOsm). The external bath solution contained 142 mM NaCl, 8 mM KCl, 1 mM CaCl_2_, 6 mM MgCl_2_, 10 mM glucose and 10 mM HEPES, calibrated to pH 7.4 with NaOH; 325 mOsm. Sodium and potassium ion currents were elicited by depolarizing voltage steps in increments of 10 mV from a holding potential of -70 to 40 mV. Miniature post-synaptic currents (mPSCs) were acquired at a holding potential of -70 mV in voltage clamp mode. Spontaneous and evoked action potentials were recorded in current-clamp mode.

**Multi-Electrode Array (MEA) recordings.**

Five MEA chambers (60MEA200/30iR-Ti-gr, Multichannel Systems) were coated with Poly-D-lysine (PDL, 1 mg/ml stock, 50 µl per electrode area) and incubated overnight at 37°C. The arrays were washed three times with sterile ddH2O and dried. Laminin (SigmaAldrich, L2020) was mixed in BrainPhys™ Media (0.05 mg/ml), added to the electrode area (50 µl) and incubated overnight at RT. After eight days of differentiation, stem cell-derived neurons were dissociated by Accutase seeded on each MEA chip (200,000 cells per MEA in total). The cells were cultured in conditional medium including 75% BrainPhys™ Neuronal Medium (BrainPhys™ Neuronal Medium supplemented with 1% penicillin-streptomycin, NeuroCult™ SM1 Neuronal Supplement, N2 Supplement-A, 20 ng/ml recombinant Human BDNF, 20 ng/ml recombinant Human GDNF and 200 nM ascorbic acid) and 25% astrocyte medium (DMEM plus N2 Supplement, 10 % One Shot™ Fetal Bovine Serum and 1% penicillin-streptomycin) which was collected from cultured rat primary cortical astrocytes. Half of the media was exchanged by fresh Media every seven days.

Spontaneous neural network activity was measured using MEA1060-Inv-BC (sampling rate 25K Hz) and software user interface (MC_Rack) provided by Micro Channel Systems (MCS). The spontaneous activity of the differentiated neural networks was measured at day 10, 15, 20, 25 and 30 post differentiation.

**MEA data analysis.**

Recorded data was replayed, and filtered (Butterworth 2^nd^ order, high pass filter cut-off at 100 Hz) and timestamps of the action potential were detected by a negative threshold (−5 standard deviation of the peak-to-peak noise). The shape and timing of the action potential were also considered. Electrodes that recorded more than three action potentials per minute (0.05 Hz) were considered as active electrodes and included in the statistical analysis. Action potential frequency was analyzed for each MEA at different time points of neuronal differentiation. The average spike frequency (action potentials per second) was measured. Action potential frequency differences at different time points were statically tested for significance (Kruskal–Wallis test). For all analyses, p < 0.05 was considered as significant.

[1] Anderson EN, Gochenaur L, Singh A, Grant R, Patel K, Watkins S et al (2018) Traumatic injury induces stress granule formation and enhances motor dysfunctions in ALS/FTD models. Hum Mol Genet 27:1366-81. <https://doi.10.1093/hmg/ddy047>

[2] Cox J, Mann M (2008) MaxQuant enables high peptide identification rates, individualized p.p.b.-range mass accuracies and proteome-wide protein quantification. Nat Biotechnol 26:1367-72. <https://doi.10.1038/nbt.1511>

[3] DeVorkin L, Gorski SM (2014) LysoTracker staining to aid in monitoring autophagy in Drosophila. Cold Spring Harb Protoc 2014:951-8. <https://doi.10.1101/pdb.prot080325>

[4] Dreser A, Vollrath JT, Sechi A, Johann S, Roos A, Yamoah A et al (2017) The ALS-linked E102Q mutation in Sigma receptor-1 leads to ER stress-mediated defects in protein homeostasis and dysregulation of RNA-binding proteins. Cell Death Differ 24:1655-71. <https://doi.10.1038/cdd.2017.88>

[5] Jesse CM, Bushuven E, Tripathi P, Chandrasekar A, Simon CM, Drepper C et al (2017) ALS-Associated Endoplasmic Reticulum Proteins in Denervated Skeletal Muscle: Implications for Motor Neuron Disease Pathology. Brain Pathol 27:781-94. <https://doi.10.1111/bpa.12453>

[6] Marrone L, Poser I, Casci I, Japtok J, Reinhardt P, Janosch A et al (2018) Isogenic FUS-eGFP iPSC Reporter Lines Enable Quantification of FUS Stress Granule Pathology that Is Rescued by Drugs Inducing Autophagy. Stem Cell Reports 10:375-89. <https://doi.10.1016/j.stemcr.2017.12.018>

[7] Naujock M, Stanslowsky N, Bufler S, Naumann M, Reinhardt P, Sterneckert J et al (2016) 4-Aminopyridine Induced Activity Rescues Hypoexcitable Motor Neurons from Amyotrophic Lateral Sclerosis Patient-Derived Induced Pluripotent Stem Cells: 4AP rescues ALS iPSC derived motor neurons. STEM CELLS 34:1563-75. <https://doi.10.1002/stem.2354>

[8] Rappsilber J, Ishihama Y, Mann M (2003) Stop and go extraction tips for matrix-assisted laser desorption/ionization, nanoelectrospray, and LC/MS sample pretreatment in proteomics. Anal Chem 75:663-70.

[9] Vizcaino JA, Deutsch EW, Wang R, Csordas A, Reisinger F, Rios D et al (2014) ProteomeXchange provides globally coordinated proteomics data submission and dissemination. Nat Biotechnol 32:223-6. <https://doi.10.1038/nbt.2839>
