# Supplementary material for: FUS pathology in ALS is linked to alterations in multiple ALS-associated proteins and rescued by drugs stimulating autophagy
Source: Acta Neuropathol. 2019 Apr 1;138(1):67–84. doi: 10.1007/s00401-019-01998-x (PMC6570784; doi:10.1007/s00401-019-01998-x)
Supplement: Supplementary file 2 — Supplementary material 2 (DOCX 61926 kb) [file 401_2019_1998_MOESM2_ESM.docx]

FUS pathology in ALS is linked to alterations in multiple ALS-associated proteins and rescued by drugs stimulating autophagy

**Authors:** Lara Marrone^1^, Hannes C.A. Drexler^2^, Jie Wang^3^, Priyanka Tripathi^4^, Tania Distler^1^, Patrick Heisterkamp^1^, Eric Nathaniel Anderson^5,6^, Sukhleen Kour ^5,6^, Anastasia Moraiti^1^, Shovamayee Maharana^3^, Rajat Bhatnagar^7^, T. Grant Belgard^7†^, Vadreenath Tripathy^1^, Norman Kalmbach^8^, Zohreh Hosseinzadeh^1^, Valeria Crippa^9^, Masin Abo-Rady^1^, Florian Wegner^8^, Angelo Poletti^9^, Dirk Troost^10^, Eleonora Aronica^10^, Volker Busskamp^1^, Joachim Weis^4^, Udai Bhan Pandey^5,6,11^, Anthony A. Hyman^3^, Simon Alberti^3^, Anand Goswami^4^ and Jared Sterneckert^1*^

**Affiliations:**

^1^Center for Regenerative Therapies Dresden, Technische Universität Dresden, Fetscherstr. 105, 01307 Dresden, Germany.

^2^Max Planck Institute for Molecular Biomedicine, Bioanalytical Mass Spectrometry, Röntgenstr. 20, 48149 Münster, Germany.

^3^Max Planck Institute of Molecular Cell Biology and Genetics, Pfotenhauerstr. 108, 01307 Dresden, Germany.

^4^Institute of Neuropathology, RWTH Aachen University Hospital, Pauwelsstr. 30, 52074, Aachen, Germany.

^5^Department of Pediatrics, Division of Child Neurology, Children's Hospital of Pittsburgh, University of Pittsburgh School of Medicine, Pittsburgh, PA, United States.

^6^Department of Human Genetics, University of Pittsburgh Graduate School of Public Health, Pittsburgh, PA, United States.

^7^Verge Genomics, San Francisco, CA, United States.

^8^Department of Neurology, Hannover Medical School, Carl-Neuberg-Str. 1, 30625 Hannover, Germany

^9^Dipartimento di Scienze Farmacologiche e Biomolecolari, Centre of Excellence on Neurodegenerative Diseases Università degli studi di Milano, Milan 20133, Italy.

^10^Amsterdam UMC, University of Amsterdam, department of (Neuro)Pathology, Amsterdam Neuroscience, Amsterdam, the Netherlands.

^11^Department of Neurology, University of Pittsburgh School of Medicine, Pittsburgh, PA, United States.

*To whom correspondence should be addressed. Email: jared.sterneckert@tu-dresden.de, Phone: +49 351 458 82103, Fax: +49 351 458 82119.

†Current address: The Bioinformatics CRO, Niceville, FL, United States.

**Acta Neuropathologica Online Resource 2: Supplementary Figures**

**Supplementary Fig. 1. Human FUS lumbar spinal cord and motor cortex histology.** **a**) R521C patients exhibit reduced numbers of lumbar spinal cord α-MNs, as demonstrated by hematoxylin immunohistochemistry (IHC) on paraffin sections. Scale bar = 200 µm. **b**) Representative DAB-IHC in lumbar spinal cord α-MNs and cortical neurons from FUS-ALS patients for FUS immunoreactivity. Scale bar = 20 µm.

**Supplementary Fig. 2. iPSC-derived neurons used in this study are electrophysiologically functional.** Untargeted iPSC-derived neurons were used in all panels. **a**) Representative voltage-gated sodium inward and potassium outward currents of a neuron recorded in voltage-clamp mode by stepwise depolarizations in 10 mV increments from a holding potential of -70 to 40 mV. **b**) As illustrated in the current–voltage plot, sodium inward currents (I_Na_) activate at -50 to -40mV, whereas potassium outward currents (I_K_) activate at -40 to -30 mV. Ion currents were normalized for neuronal sizes based on the capacitance of the cell membrane (pA/pF). n=25. All values are presented as mean ± SEM. **c**) Recording in voltage-clamp mode reveals the occurrence of spontaneous miniature postsynaptic currents (72.0% of investigated cells) as synaptic activity. **d**) Spiking of multiple spontaneous action potentials (39.1% of investigated cells). **e**) Neuron firing repetitive action potentials upon depolarization in the current-clamp mode (17.4% of investigated cells). **f**) Representative image of a multielectrode array (MEA) chamber with iPSC-derived neurons. **g**) A representative digitized voltage trace of responsive electrode is shown. **h**) A recording of selected action potential is shown. **i**) Rastergram of 40 electrodes from neurons using MEA. Each spot indicated an action potential over 30s recordings. **j**) Average action potential frequencies recorded from stem cell-derived neuronal cultures at the indicated days of differentiation. n = 5 MEAs per time point, **<0.01 according to Kruskal–Wallis test.

**Supplementary Figure 3. Overview of the differentiation protocol used in this study and characterization of obtained cell populations.** **a**) Schematic representation of the differentiation steps. **b**) Immunofluorescence staining of small molecule-derived neural progenitors (smNPCs) confirms expression of neural progenitor markers. **c**) Immunofluorescence for neuronal markers confirms generation of neurons. Double-positivity for Islet1 and HB9 identifies bona fide MNs. **d**) Frequency of Islet1- and HB9-positive cells. **e**) Characterization of marker expression following RNA-sequencing reveals the neuronal subtypes populating our iPSC-derived neuronal cultures. **f**) Eigengene classification confirms the presence of spinal cord neurons, with a small percentage of MNs.

**Supplementary Figure 4. Investigation of autophagy markers in iPSC-derived neurons.** **a**) WES capillary electrophoresis and respective quantification of FUS-eGFP and p62 proteins in LL iPSC-derived neurons. Combined inhibition of autophagy and proteasome activity via 3-MA and MG132 increases FUS levels in P525L LL neurons and triggers upregulation of p62 in both WT and P525L LL neurons, with mutant neurons exhibiting the highest levels. **b**) Confocal micrographs showing the indicated cell lines subjected to the specified treatments. **c**) Quantification of panel (**b**) for cytoplasmic FUS-eGFP. **d**) Confocal micrographs displaying P525L SL neurons following sodium arsenite treatment. Arrow heads identify colocalization of FUS-eGFP and p62 foci. Scale bar=10 µm. **e**) Immunoblot for LC3 levels in iPSC-derived neurons treated with 50 µM chloroquine for 24h. LC3-II increases in all neurons with mislocalized FUS. Immunoblot for (**f**) LC3 and (**g**) Beclin-1 levels following treatment with different mTOR inhibitors. Torkinib is the best autophagy stimulator. n=3. Error bars indicate standard deviation. *, **, ***, and **** correspond to p < 0.05, 0.01, 0.001, and 0.0001, respectively.

**Supplementary Figure 5. Changes in ALS-associated protein levels are specific to neurons. a**) Western blot showing the investigated ALS proteins in SL lines across differentiation. **b**) Quantification of p62 protein levels. **c**) Quantification of EWSR1, TAF15, hnRNPA1 and hnRNPA2B1 protein levels. Results show that significant changes are limited to P525L neurons. n=3. Error bars represent S.E.M. * corresponds to p < 0.05.

**Supplementary Figure 6. Immunohistochemistry of human tissue.** DAB-IHC for (**a**) EWSR1 and (**b**) TAF15 in the spinal cord of FUS-ALS patients and controls. Black arrows identify protein aggregates; white arrow heads pinpoint RBP nuclear depletion. Double immunofluorescence labelling for FUS and (**c**) EWSR1 or (**d**) TAF15. Red arrow heads show colocalization of FUS and the investigated RBPs in proteinaceous inclusions.

**Supplementary Figure 7. Generation of knockdown vectors.** **a**) Schematic representation of the knockdown constructs used in this study. Fragments of pre-existing vectors were excised and re-ligated in the presence of the U6 promoter and shRNA sequence of interest via Gibson assembly. The “empty vector” lacked the U6+shRNA fragment. **b**) *in silico* prediction of the band pattern resulting from restriction digestion of the putative knockdown construct using KpnI+PvuI (above) or KpnI+NdeI (below). Cutting sites for these enzymes are shown in (**a**). Presence of the U6 promoter sequence causes a change in the band pattern. **c**) Agarose gel images reporting the digestions described in (**b**). L = 1 kb ladder, E = empty vector. Major differences are pointed out by arrow heads.

**Supplementary Figure 8. Validation of knockdown vectors.** **a**) Sanger sequencing results confirm correct shRNA sequence insertion for all produced knockdown vectors. **b**) Validation of RBP knockdown efficiency in HEK293T cells transfected with the generated constructs. n=3. Error bars indicate standard deviation. * and ** correspond to p < 0.05 and 0.01, respectively.

**Supplementary Figure 9. Lentivector dilution and infection duration titration.** **a**) Bar graphs represent the percentage of infected cells upon neuronal infection using the indicated dilutions. Quantification assesses the number of tdTomato-positive cells over total cell numbers. **b**) Bar graphs show the amount of infected neurons at day 9, 6, 3, and 1 post-infection. Quantification evaluates the abundance of tdTomato fluorescent signal per image. Results are reported for all produced lentivectors, including constructs A, B, and C for each EWSR1, TAF15, hnRNPA1, and hnRNPA2B1, as well as for the empty vector.

**Supplementary Figure 10. mTOR inhibition in iPSC-derived neurons enhances protein clearance and restores proteostasis. a**) Quantification and exemplary confocal micrographs of stress granule size in neurons stressed with sodium arsenite (NaSaO2) in the presence or absence of Torkinib and PQR309. Treatment with these mTOR inhibitors reduces the amount of cytoplasmic FUS-eGFP protein available for recruitment to SGs. Scale bar=25 µm. **b**) Filter retardation assay showing increased propensity for aggregation in P525L SL neurons compared to WT. Autophagic clearance of cytoplasmic P525L FUS by torkinib or PQR309 ameliorates the phenotype. **c**) PQR309 restores physiological levels of RNA-binding proteins which are downregulated in SL P525L neurons. n=3. Error bars indicate standard error of the mean (S.E.M). *, ** and **** correspond to p < 0.05, 0.01, and 0.0001 respectively.
